# A six gene expression signature defines aggressive subtypes and predicts outcome in childhood and adult acute lymphoblastic leukemia

**DOI:** 10.18632/oncotarget.4113

**Published:** 2015-05-12

**Authors:** Jin Wang, Jian-Qing Mi, Alexandra Debernardi, Anne-Laure Vitte, Anouk Emadali, Julia A. Meyer, Konstantina Charmpi, Bernard Ycart, Mary B. Callanan, William L. Carroll, Saadi Khochbin, Sophie Rousseaux

**Affiliations:** ^1^ State Key Laboratory for Medical Genomics and Department of Hematology, Shanghai Institute of Hematology, Collaborative Innovation Center of Systems Biomedicine, Pôle Sino-Français des Sciences du Vivant et Genomique, Rui Jin Hospital, Shanghai Jiao Tong University School of Medicine, Shanghai, China; ^2^ INSERM, Université Grenoble Alpes, Institut Albert Bonniot, Grenoble, France; ^3^ NYU Cancer Institute, NYU Langone Medical Center, New York, USA; ^4^ Laboratoire Jean Kuntzmann, CNRS, Grenoble, France

**Keywords:** cancer, personalized medicine, risk stratification, minimum residual disease, cancer stem cells

## Abstract

Abnormal gene expression in cancer represents an under-explored source of cancer markers and therapeutic targets. In order to identify gene expression signatures associated with survival in acute lymphoblastic leukemia (ALL), a strategy was designed to search for aberrant gene activity, which consists of applying several filters to transcriptomic datasets from two pediatric ALL studies. Six genes whose expression in leukemic blasts was associated with prognosis were identified:three genes predicting poor prognosis (*AK022211, FASTKD1* and *STARD4*) and three genes associated with a favorable outcome (*CAMSAP1, PCGF6* and *SH3RF3*). Combining the expression of these 6 genes could successfully predict prognosis not only in the two discovery pediatric ALL studies, but also in two independent validation cohorts of adult patients, one from a publicly available study and one consisting of 62 newly recruited Chinese patients. Moreover, our data demonstrate that our six gene based test is particularly efficient in stratifying *MLL* or *BCR.ABL* negative patients. Finally, common biological traits characterizing aggressive forms of ALL in both children and adults were found, including features of dormant hematopoietic stem cells, suggesting new therapeutic strategies.

## INTRODUCTION

Cell differentiation involves waves of successive gene expression programs driving progenitor cells through various developmental stages to finally reach a characteristic stable pattern of gene expression in fully differentiated adult cells. One of the remarkable characteristics of malignant cells is their inability to maintain gene silencing including that of tissue-specific genes [1]. This reflects the necessity of cancer cells to acquire new properties, which are absent in the normal cells of origin [2]. Aberrant gene expression could provide cells with a whole range of new properties required to reach the state of malignancy [3], as well as to develop resistance to therapeutic drugs [4]. This hypothesis implies that established malignant cells should be highly dependent on the expression of such genes and that the expression of a subset of these genes may be associated with their clinical behavior [1, 3, 5-8]. Based on this hypothesis, we explored the gene expression profiles of acute lymphoblastic leukemia (ALL) cases in both children and adults in order to systematically detect aberrant expression of certain genes that could have value as prognosis markers.

While the outcome for children with ALL has improved, the survival for those who experience a relapse remains dismal. Likewise, adults have an inferior outcome compared to children [9]. A variety of clinical (e.g., age and gender), and laboratory (e.g., white blood cell count, blast genotype and initial response to therapy) variables have been identified that correlate with outcome. Adjusting treatment based on these characteristics (so called risk stratification) has enabled treatments to be intensified in some patients to improve outcome, while avoiding unnecessary dose escalation for those with a favorable prognosis. However, in spite of this progress, relapses still occur in patients initially classified as low risk, prompting the search for additional variables to refine risk stratification. Moreover identifying biological pathways that are responsible for transformation and therapy resistance will allow the development of targeted approaches thereby avoiding the collateral side effects of conventional agents.

The definition of a new test based on the activation of 6 genes and its outcome in terms of patient stratification reported here enabled us to identify the most aggressive forms of ALL in both children and adults, independently from other known prognostic parameters in use in the clinic, such as *MLL* or *BCR.ABL* translocations. It also led us to highlight specific biological properties of these aggressive ALL forms, including their enrichment in quiescent non-dividing stem cell-like functions/properties.

## RESULTS

### Identification of distinctive ALL transcriptional features

**Figure 1:**
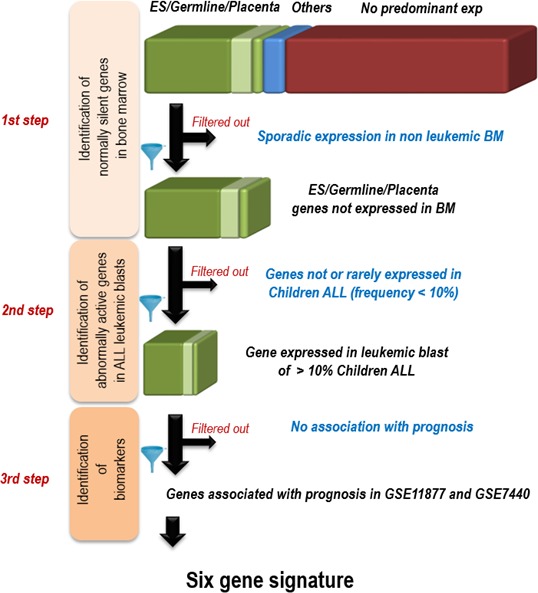
Strategy for the identification of 6 genes whose activation in pediatric ALL bone marrow is associated with prognostic 1st step: identification of normally silent genes in bone marrow; The predominance of expression was defined according to the distribution of expressions in a large set of normal human tissues (found on the Gene Expression Omnibus (GEO) website ((http://www.ncbi.nlm.nih.gov/geo/): references GSE3526 for adult tissues, GSE7434, GSE9994 and GSE18809 for placenta, GSE11350 for Embryonic stem (ES) and male germ cells). A gene was considered predominantly expressed in one tissue when its expression level in this tissue was above the mean expression in all tissues + 3 standard deviations; Using available transcriptomic data from 73 non-leukemic bone marrow samples from GSE13204, the genes showing some expression in non-leukemic bone marrow samples were filtered out 2nd step: selection of genes expressed in > 10% of pediatric ALL; Using transcriptomic data of pediatric ALL made publicly available by [12] (GEO reference GSE11877) and by [13] (GSE7440), genes aberrantly expressed in less than 10% of tumors were filtered out; 3rd step: selection of genes associated with prognostic using the survival data of the same studies (see Supp. Figure 1).

The specific contribution in oncogenesis of genes normally silent in most tissues but expressed in limited number of cell types such as Embryonic Stem (ES) cells, placenta or male germline cells, has been demonstrated by our previous work, as well as by data from the literature [1, 3-8, 10]. Therefore, we first selected genes predominantly expressed in ES cells, placenta or male germline cells (Figure 1 step 1), following a strategy based on the analysis of available transcriptomic data. We found 10471 ES/placenta/germline predominant genes. Among them, those sporadically expressed in normal adult bone marrow were filtered out, leaving a list of 7244 genes, which could be considered as normally silent in healthy bone marrow. The approach is detailed in Material and Methods.

The second step consisted of looking for the aberrant expression of these genes in ALL samples. In order to do so, for each gene, a threshold between background levels and expression of the gene had to be established. Following an approach previously used and validated [1], based on the fusion of uniformly normalized independent Affymetrix transcriptomic datasets, we considered a signal as positive if it was above a threshold equal to the mean + 3 standard deviations of its expression values in 112 normal somatic adult tissues. We screened transcriptomic data of pediatric ALL patients from two publicly available series, both available on the GEO database under the respective references of GSE11877 [11, 12] and GSE7440 [13], that were obtained using the same Affymetrix technology as the normal tissue data. Of the 7244 ES/placenta/germline predominant genes defined as normally silent in bone marrow, the expression of 2119 were above the threshold (defined above) in the blasts from blood or bone marrow of at least 10% of pediatric ALL cases of both series of patients (Figure 1 step 2).

### The expression of six genes is significantly associated with prognosis in pediatric ALL

We then explored the potential of these genes for possible clinical significance. For this purpose, we took advantage of the availability of follow-up data in the two above-mentioned childhood ALL series. For each of the 2119 genes expressed in more than 10% of the cases of the children with ALL, we compared the survival probabilities of the patients whose blasts expressed the gene with those whose blasts did not. For each gene, hazard ratios (HR) for the association with the overall survival probability were calculated independently in both settings. Out of the 2119 genes, 416 were consistently associated with shorter survival in both studies (HR > 1), whereas 693 were consistently associated with longer survival in both studies (HR < 1). Respectively 6 and 17 out of the genes negatively or positively associated with prognosis had a logrank test *p*-value < 0.05 in both studies. We ranked these genes by increasing *p*-values and selected the first three of each list (Figure 1 step 3; Supp. Figure S1). These include 3 genes, *CAMSAP1, PCGF6* and *SH3RF3*, whose aberrant expression was significantly associated with a higher probability of long-term survival (called hereafter “positive genes”) and another 3 genes, *AK022211, FASTKD1* and *STARD4*, whose expression was associated with shorter survival (named hereafter “negative genes”) (Supp. Table S1).

Considering the expression of the six genes in large groups of normal tissues, including germline tissues, embryonic stem (ES) cells, placenta and adult somatic tissues, we confirmed that the maximum expression levels of these genes were in germline cells, ES cells and/or placenta, and the lowest expression levels were largely confined to adult somatic tissues (Supp. Figure S2). In order to address their expression during normal hematopoiesis more specifically, we also examined their levels of expression using available data from specific stages and types of hematopoietic cells (Supp. Figure S3). Whereas *CAMSAP1* was not highly expressed at any stages of hematopoiesis, the other genes had relatively high expression levels in at least one specific hematopoietic cell type/stage. Indeed *AK022211, FASTKD1* and *PCGF6* are active in early precursors of hematopoietic cells (CD34 positive cells from cord blood and/or bone marrow), while *STARD4* was found to be preferentially expressed in blood B-lymphocytes and *SH3RF3* in bone marrow neutrophils.

### Combinations of genes whose expression is associated with significant differences in survival probabilities led to the establishment of a prognosis classification tool for childhood ALL

**Figure 2:**
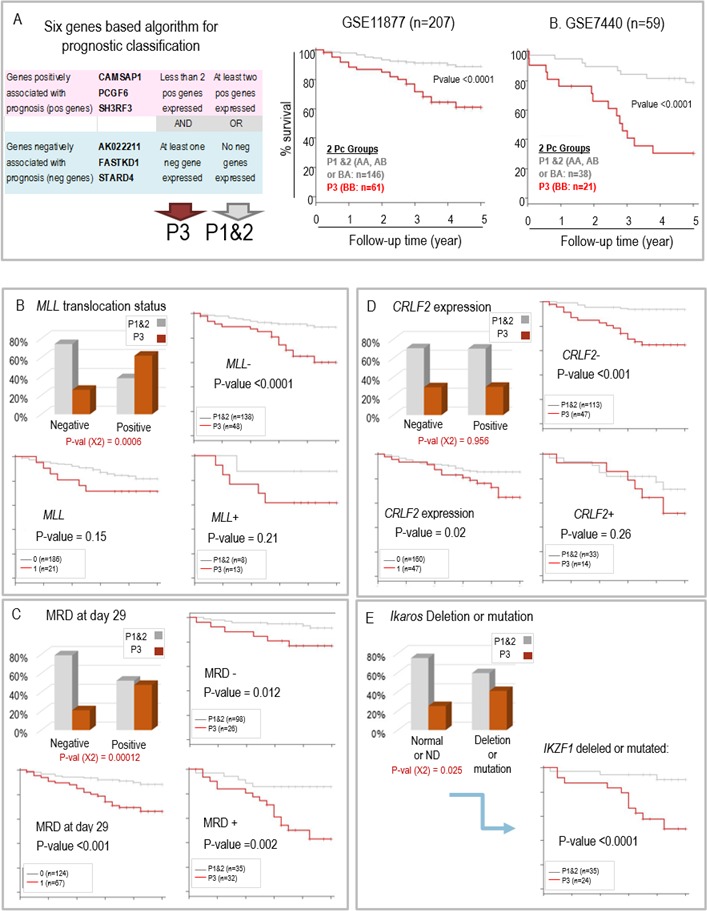
**A.** Cumulative global Kaplan Meyer overall survival estimates comparing the ALL patients from the GSE11877 (middle panel) and GSE7440 (right panel) after the final prognostic classification. Tumors in both GSE series were stratified into P1&2 and P3 group as a function of the expression of the 6 genes classifier following the algorithm shown in the left panel. **B.** to **C.** Prognosis groups and bio-clinical data in children ALL (GSE11877): **B.** MLL status; **C.** Minimal residual disease at day 29; **D.** CRLF2 expression; **E.** IKAROS gene deletion or mutation; in **B.**, **C.** and **D.,** the histogram panel (upper left) shows the respective proportions of the two prognostic groups P1&2 and P3 in the patients with ALL negative or positive for the indicated molecular characteristic, the Kaplan Meier survival curves compare survival probabilities between the patients according to their molecular diagnostic (lower left panel) or between the two prognosis groups (P3 versus P1&2) within the patients whose ALL is negative (upper right panel) or positive (lower right panel) for the molecular characteristic.

Based on the selected six genes we designed an algorithm to stratify childhood ALL. A detailed presentation of survival probabilities in ALL patients with blasts expressing different combinations of “negative” and “positive” genes is shown in Supp. Figure S4. In our “learning group” (GSE11877), an optimized separation of patients was obtained as follows: patients with blasts expressing one or more of the “negative genes” associated with none or only one of the “positive genes” had the shortest survival probability (called here P3). In contrast, patients with blasts expressing none of the “negative genes” and at least two of the “positive genes” would fall within a group with the most favorable prognosis (called here P1). An intermediary group, P2, comprised patients either expressing at least one of the “negative genes” together with at least two “positive genes” or expressing less than two “positive genes” but none of the “negative genes”. Since survival probabilities were not significantly different between patients of the P1 and P2 groups, based on the active expression of the 6 genes found in their blasts, the patients were finally assigned to two main groups, P1&2 (high probability of long term survival) and P3 (low survival probability) (Figure 2A left panel).

Hence, this algorithm enabled the identification of 61 cases (29%) of ALL from this series (GSE11877) with highly reduced survival probabilities (Figure 2A, middle panel). The same algorithm used in the second series of ALL patients (GSE7440) confirmed the potential of this combination of genes to identify aggressive ALL, corresponding to 21 cases (35%) in this series of patients (Figure 2A, right panel).

### A six-gene classifier has stronger prognostic value than several known ALL prognostic factors

By comparing the distribution of our prognosis groups and clinical and laboratory data of pediatric ALL patients from the series GSE11877 we found that P3 ALL were enriched in some, but not all, of the poor prognosis ALL clinical and molecular categories. Interestingly, several of the factors which have been described previously as associated with poor prognosis in ALL patients, including the presence of blasts in the central nervous system (CNS disease), gender, age > 10 years old, or > 50G white blood cells (WBC) at diagnosis (Supp. Figure S5), or even the presence of a *MLL* translocation (Figure 2B: lower left panel) were not associated with significantly shorter survival in this particular series of patients. It should be stressed here that the patients selected for this study were all identified as high-risk B-precursor ALL patients, meaning that they belonged to categories of patients associated with an inferior outcome in previous studies [12]. Therefore, they all had at least one of the risk factors including central nervous system disease or age (10 years old or older) or high WBC count.

However, other known clinical and biological parameters associated with poor prognosis, including minimum residual disease (MRD) detected at day 29 of treatment (Figure 2C), or a high *CRLF2* expression level (Figure 2D) were correlated with shorter survival in the present study (Figure 2C and 2D: lower left panels). The *MLL* translocation positive subgroup and MRD positive cases showed enrichment in P3 forms, but *CRLF2* did not (Figure 2B, 2C and 2D: histograms in upper left panels).

In a univariate approach, we assessed the prognostic value of our new classifying six genes algorithm with respect to that of these factors. Considering each of the following parameters (MLL translocation, MRD at day 29 and CRLF2 overexpression), we found that, within each subgroup of patients which could be defined according to their status, our 6 gene classifier could also separate the patients into two prognosis classes, and therefore enabled refining the prognosis (Figure 2B, 2C and 2D: right panels). For instance, in this series of GSE11877 patients *CRLF2* expression level could separate two groups of patients with significantly different survival probabilities (Figure 2D, left lower panel). However, among *CRLF2* low expressing patients (*n* = 160), our classifying genes enabled to isolate a subgroup of 47 patients with a much poorer prognosis, whereas among *CRLF2* high expressing patients (*n* = 47), it could differentiate two groups of higher and lower survival probabilities (*n* = 33 and *n* = 14 respectively) but with no statistical significance (Figure 2D, right panels). Additionally, deletion or a mutation of the *IKZF1* gene, also a factor associated with poor prognosis in many studies, was previously identified in 59 patients of the GSE11877 cohort (Supp. Table S8 in [14]). By classifying these 59 patients into prognosis groups using our six genes, we could clearly isolate a group of patients with good prognosis (35 patients, most remaining alive after 5 years), whereas approximately 40% of the 27 P3 patients survived after 5 years (Figure 2E). Altogether, these data show that our six-gene classifier increases our ability for outcome prediction, particularly in *MLL* negative patients, MRD positive cases, CRLF2 positive or IKZF1 deleted or mutated patients. This observation is also illustrated by the forest plot presented Supp. Figure S6.

The specific contribution of our 6-gene classification system as an explanatory variable of survival probability was also tested in a multivariate analysis including all these parameters, which clearly demonstrated that our six gene-prognostic test is the most significant explaining variable (Supp. Table S2).

### The six gene-based algorithm can predict outcome in adult ALL

We questioned whether this six-gene classification algorithm could also be applied to predict outcome of ALL in adult patients. This question was addressed by analyzing the predictive power of our six-gene combination using two publicly available datasets of adult ALL.

**Figure 3:**
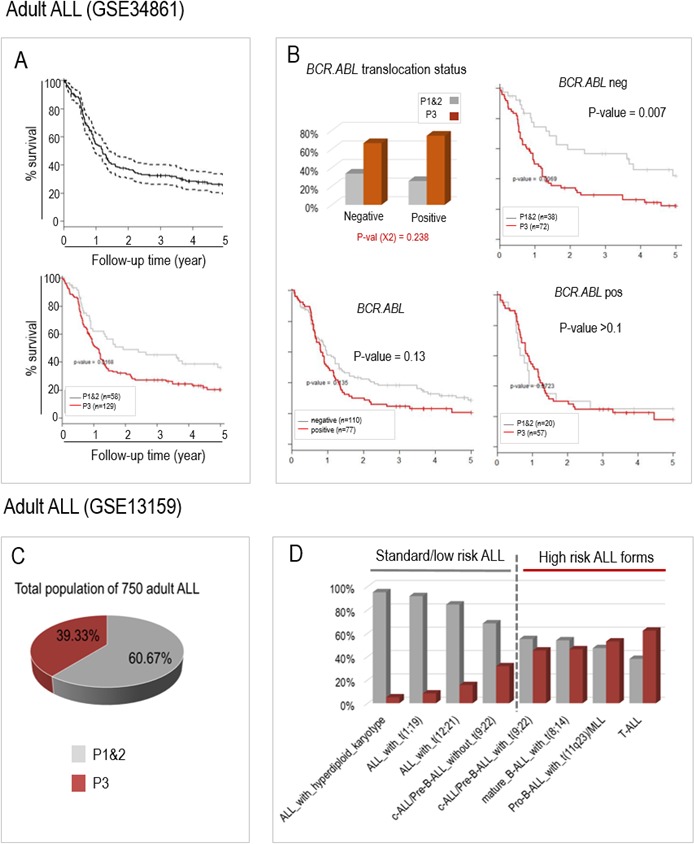
The aberrant activation of the same 6 genes can also predict prognosis in two published populations of adult ALL patients from GSE34861 (A and B) and GSE13159 (C and D, MILE study) **A.** Survival probabilities of adult ALL patients from GSE34861 study can be predicted by our 6 genes classifying system; The upper panel shows the global survival probability of the whole 187 patients of this published series (GSE34861), and the lower panel shows the survival probabilities of the patients assigned to the “P1&2” (grey curve, *n* = 58) and to the “P3” groups (red curve; *n* = 129). **B.** Our 6 genes classification system predicts aggressive ALL among the t(9;22) (BCR.ABL) negative patients. As in Figure 2B-2D, the histogram panel (upper left) shows the respective proportions of the two prognostic groups P1&2 and P3 in the patients with ALL negative or positive for the *BCR.ABL* translocation, the Kaplan-Meier survival curves compare survival probabilities between the patients according to the presence or absence of BCR.ABL translocation (lower left panel) or between the two prognosis groups (P3 versus P1&2) within the patients whose ALL is negative (upper right panel) or positive (lower right panel) for BCR.ABL translocation. **C.** and **D.** In the 750 ALL patients from the “MILE” American study (GSE13159), the known aggressive molecular subtypes of adult ALLs co-segregate with the “P3” ALL. C. The pie chart shows the distribution of P1&2 patients (60%) and P3 patients (40%). D. The histograms show the relative proportions of P1&2 and P3 patients for each molecular subtype of ALL. The molecular subtypes are ordered from the less to the most aggressive known forms (left to right).

First, we explored the predictive ability of the algorithm using an American publicly available series of 187 B-ALL patients with bone marrow transcriptomic data (GSE34861 on GEO website) and for which survival data were available. By using exactly the same approach and the same six genes, the patients were assigned to two groups corresponding to P1&2 and P3 respectively, and their survival probabilities were compared. As shown in Figure 3A (lower panel), the survival of the 129 adult P3 patients was significantly shorter than the survival of P1&2 patients, suggesting that the 6-gene classification algorithm is also able to identify aggressive forms of ALL in adult patients. Moreover, within this cohort of B-ALL, 77 patients were positive for the translocation t(9;22) (*BCR.ABL*), and those had a shorter survival probability than the remaining 110 *BCR.ABL* negative patients (although the difference did not reach statistical significance). Interestingly, our 6-gene algorithm was very efficient in identifying aggressive forms of ALL, among the 110 *BCR.ABL* negative patients (Figure 3B).

Second, using a larger transcriptomic data set from 750 adult ALL patients (of various molecular subtypes) which was part of the American “MILE” study (GEO website: GSE13159), the same 6 gene-based algorithm was used again to classify these adult ALL into P1&2 and P3 groups. No survival data were available for this series of adult ALL patients but useful information was provided about their molecular subtypes (also available from GEO website). We measured the enrichment of the different molecular subgroups of adult ALL cases in our two prognosis groups P1&2 and P3, obtained with our 6 genes. For this purpose, we took into account the already established survival data of the different subtypes of adult ALL [15, 16]. As shown in Figure 3C and 3D, the distribution of the patients into P1&2 and P3 groups respectively co-segregated well with ALL subtypes known to correlate with a better or worse prognosis (also see Supp. Table S3). Indeed, patients with high risk ALL forms, such as those with *MLL* or *BCR.ABL* translocations, were preferentially assigned to our “P3” poor prognosis group with the 6 gene classifier, whereas patients with better prognosis forms of ALL (standard or low risk ALL), such as those with ETV6/RUNX1 or hyperdiploid karyotypes, where mostly classified “P1&2”.

### Aggressive pediatric ALL are characterized by a transcriptomic profile, which is partially overlapping with adult ALL

**Figure 4:**
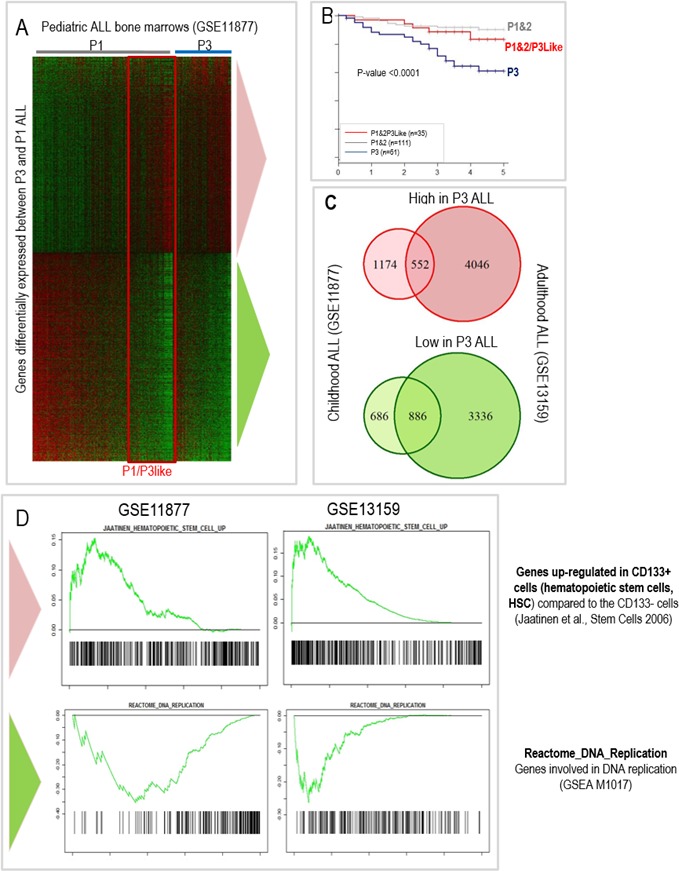
The blasts transcriptomic and functional signatures characterizing the « P3 » group of aggressive ALLs partially overlap between children (GSE11877) and adult (GSE13159) patients **A.** Heatmap showing the genes significantly upregulated (upper part of the heatmap) or down regulated (lower part of the heatmap) in the bone marrows of P3 ALL as compared to P1&2 children ALL (GSE11877). Low to high expression levels are represented on a green to red scale. **B.** Prognostic of “P1&2/3-like” ALL compared to other P1&2 and P3 ALL. **C.** Overlaps between transcriptomic signatures of P3 children ALL and adult ALL (from GSE13159) assigned to a P3 group using the same 6 genes classification algorithm. **D.** Gene Sets Enrichment Analysis (GSEA) of the aggressive ALL profile in pediatric ALL (GSE11877) and adult ALL patients (GSE13159) showing a highly significant overlap between the up-regulated genes in aggressive P3 ALL and hematopoietic stem cells (CD133+) (top panels; [20]) but a down-regulation of DNA replication associated genes (lower panels).

Our ability to specifically predict outcome in childhood ALL, independently from other prognostic parameters (in GSE11877), enabled us to carry out a supervised transcriptomic analysis looking for genes differentially expressed between the 61 ALL cases associated with an inferior outcome (P3) and the other cases of ALL with better prognosis (P1&2). This analysis, performed considering all the human genes represented on the 54K Affymetrix microarray, showed that 1572 genes were down regulated and 1726 up regulated in the ALLs associated with poor outcome (corrected Mann-Whitney *p* < 0.01). This expression signature characteristic of P3 ALL forms is represented in Figure 4A demonstrating striking differences between P1&2 and P3 patients for these genes. However a subset of P1&2 patients (*n* = 35) showed a gene expression profile very similar to the P3 ALL (Figure 4A: red rectangle). Interestingly, the survival probability of the corresponding patients group with this “P3-like” profile was close to that of the P1&2 patients (Figure 4B), demonstrating firstly that these patients had been correctly assigned to the good prognosis group by our 6 gene based classification algorithm, and secondly that the P3 gene expression profile, is not sufficient to define the ALL aggressiveness.

Despite the overall similarities in the gene expression profile, we have looked for genes differentially expressed between these two ALL populations, P3 versus P1&2 “P3-like”, in order to better delineate the aggressive phenotype of P3 tumors. 27 genes had a significant increase of their expression levels in P3 ALL blasts compared to P3-like blasts and, in contrast, 2 other genes showed a decreased expression (listed in Supp. Table S4). A close inspection of these genes revealed additional properties of the P3 ALL, and pointed to possible new targets for existing (i.e., CDK6: [17]) or to be developed (i.e., ATAD2: [18]) drugs.

Although there are important clinical and biological differences between pediatric and adult ALLs [15], we observed that the global transcriptomic profile in aggressive childhood ALL partially overlapped the global transcriptomic profile of down- and up- regulated genes in the subgroup of adult ALL patients, classified “P3” by using the same combinations of 6 genes (Figure 4C).

In order to deepen our understanding of the biological significance of our findings and highlight specific characteristics of the subgroup of aggressive ALLs, we explored functional data, including gene ontology functions enriched or depleted and the overlaps with gene expression signatures found in physiological or other pathological situations. We carried out a Gene Sets Enrichment Analysis (GSEA) on the collections of gene sets made available by the Broad Institute [19] using an approach derived from the generic GSEA analysis (Charmpi and Ycart, http://arxiv.org/abs/1410.1620). This revealed that the forms of ALL with a poor outcome, which we identified as “P3”, both in children and adult patients, activate a gene group significantly overlapping hematopoietic stem cell gene expression profiles (CD133+ cells, [20]), whereas most genes involved in proliferation and cell-cycle related functions show a decreased expression as illustrated in Figure 4D (enriched and depleted gene sets listed in Supp. Table S5 and S6). Although unexpected, this observation suggests that aggressive ALL could have acquired properties of pluripotent non-dividing stem-like cells. A close consideration of GO terms also indicates that P3 tumors harbor profound alterations of their nuclear and metabolic functions (Supp. Table S7).

### RT-qPCR based detection of our six prognostic genes predicts response to induction treatment and disease free survival probability in a prospective cohort of 62 Chinese adult ALL patients

**Figure 5:**
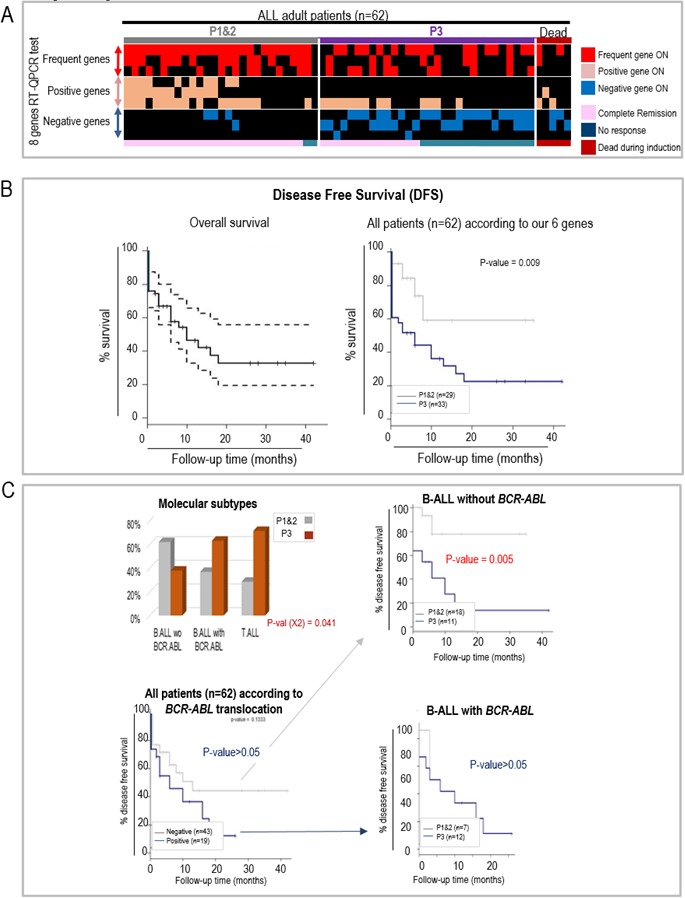
RT-qCPR detection of the aberrant expression of 6 genes helps predicting the response to an induction protocol and disease free survival (DFS) in a prospective study of Chinese adult ALL patients **A.** Heatmap illustrating the classification of ALL patients (x-axis) in the P1&2 or P3 groups using our RT-qPCR test, detecting the expression in bone marrow of 3 frequent genes (used as controls, *COX8C*, *DKFZp761D1918* and *RPL10L*), as well as the 6 prognostic genes (y-axis), including 3 genes associated with good prognosis (positive genes: *CAMSAP1, SHR3F3* and *PCGF6*) and 3 genes associated with poor prognosis (negative genes: *FASTKD1, STARD4* and *AK022211*). The patients' response to the induction therapy is indicated below the heatmap. Induction failure (no response) was defined as the persistence of leukemic blasts in the bone marrow (defined as marrow with > = 5% blasts) or leukemic blasts found at an extramedullary site at the end of the induction therapy. Complete remission was defined as marrow with < 5% blasts, and normal cellular bone marrow with trilineage hematopoiesis. **B.** Disease Free Survival (DFS) of the whole population of 62 patients (left panel: confidence interval 95%) and of the patients assigned to the P1&2 and P3 groups, using our 6 genes algorithm (RT-QPCR detection). **C.** DFS according to the translocation BCR.ABL and our six genes. As in Figure 3B, the histogram panel (upper left) shows the respective proportions of the two prognostic groups P1&2 and P3 in the patients with B-ALL negative or positive for the *BCR.ABL* translocation, and the Kaplan Meier survival curves compare survival probabilities between the patients according to the presence or absence of *BCR.ABL* translocation (lower left panel) or between the two prognosis groups (P3 versus P1&2) within the patients whose ALL is negative (upper right panel) or positive (lower right panel) for *BCR.ABL* translocation.

Finally, using RT-qPCR we validated the use of our 6 genes to help predict the response to the conventional induction therapy (Vincristine/Daunorubicin/Cyclophosphamide/Prednisone/L-Asparaginase administered over a 4 weeks period) in a series of 62 adult ALL patients (clinical data detailed in Supp. Table S7). RNA was prepared from the bone marrow cells of these patients at diagnosis and the expression of the six diagnostic genes was detected by RT-qPCR (primers listed in Supp. Table S8). Following exactly the same algorithm, these patients were assigned to either group P1&2 or P3 (Supp. Table S7). The response to the induction treatment was evaluated by the quantification of leukemic blasts in the bone marrow and MRD quantified at Day 29 by eight-color flow cytometry. Five patients died during the induction protocol and were therefore excluded from the subsequent analyses. All 11 patients assigned to the “P1” group achieved complete remission after the induction treatment, and most (14/16, 87%) patients assigned to the “P2” group also did. Considering all 18 patients that did not respond to the induction treatment, 16 (89%) had been correctly assigned to the P3 group, but two had been misclassified into the P2 group. Among the 39 patients achieving a complete remission (CR) after induction, 25 (64%) had been assigned to P1&2 prognostic group, but the other third had been assigned P3 (Figure 5A). Interestingly, a follow-up of the disease free survival (DFS) of these patients demonstrates that our six genes test can efficiently identify patients with a significantly poorer outcome (Figure 5B). As expected, the P3 group is associated with the most aggressive molecular subtypes (B-ALL with *BCR.ABL* and T-ALL). However, as observed with the GSE34861 published study, the six gene based classification was also particularly informative in the case of the B-ALL patients without the *BCR.ABL* translocation, since, for these patients, belonging to the “P3” group, means a significantly poorer prognostic than belonging to the P1&2 group (Figure 5C).

## DISCUSSION

The work presented here supports our previous observation that the aberrant expression of key genes contributes to response to therapy. Indeed, in a previous study, we demonstrated firstly that tumors of all origins activate a large number of tissue-specific genes, and secondly, in lung cancers, the activation of a subset of these genes could identify very aggressive tumors [1].

In the present report, we propose that in hematopoietic malignancies, like in solid tumors, abnormal gene expression could also be a critical determinant of cancer progression and that the malignant cells' response to therapy could correlate with the expression of a subset of critical genes.

We found that, in both pediatric and adult ALL patients, a simple algorithm based on the combined expression of 6 genes can predict the likelihood of a patient's respond to treatment and their survival probability. Indeed, these 6 genes, which were initially identified based on their strong association with survival in ALL children, could in combination not only predict survival in a cohort of adult patients, but also demonstrated a specific profile for aggressive molecular forms in another larger cohort of adult ALL. In addition, the predictive power of our six genes was re-enforced when we found that their expression status, measured by RT-qPCR, is also associated with the response of adult patients to current treatments in a prospective study of 62 adult ALL patients.

In this prospective study, we developed a RT-qPCR based assay to detect the expression of the 6 genes and demonstrated our ability to predict their response to a modified CALGB induction chemotherapy (VDLCP regimen) used as standard therapy for treatment of adult ALL today [16, 21].

In our series of 62 patients, the complete remission (CR) rate was 63%, which is slightly lower than the remission rate that we previously reported for a larger series (74.6%, [16]). Five patients died during the induction protocol, likely because of the toxicity of the treatment, and were therefore not considered in the following analyses. Using the RT-qPCR, all 11 patients that had been assigned to the good prognostic P1 group, responded to the induction protocol. Although two of the 16 “P2” patients did not respond, the “P2” group still predicted a better chance of response than the “P3” group, where more than half (16 out of 30) of the patients did not respond to the induction treatment (Figure 5A). More than a third (36%) of “good responders”, as classified by conventional prognostic criteria, were assigned to the “P3” group. However, follow-up of disease free survival of these “P3” patients over 40 months clearly indicates that they have a significantly lower disease-free survival probability than the patients assigned to the P1&2 group, suggesting that these patients define a currently cryptic poor prognosis subtype (Figure 5B).

Currently, MRD is considered as one of the most powerful indicators for prognosis evaluation in ALL, even in patients with low-risk features at presentation, especially when it turns out to be positive [22]. Our data suggest that our 6-gene based classification is a useful indicator of response, in addition to MRD for several reasons. Firstly, here we observe that 22 MRD-positive (leukemic cells above 0.01%) patients out of 31 are in the P3 group but, it is remarkable that we are able to further identify new high-risk patients within the group of MRD negative patients (*n* = 8) (Supp. Table S7). Secondly, in a small cohort of patients the MRD pattern may not be available, mainly due to technical reasons [23] and in this particular situation, our classifying method would be critical in the clinical decision-making. Thirdly, our 6-gene classification test can rapidly give an answer shortly after diagnosis, whereas MRD can only be taken into account at later time points. This allows for early intensification of therapy. Additionally, our 6-gene based prognostic test was particularly informative when applied on *BCR.ABL* negative patients, since it clearly helped identifying a subset of patients with poor outcome.

Altogether, our data strongly support the robustness of our classifying method, and its relevance in combination with other clinical and molecular characterization of ALL patients, particularly in the case of *MLL* or *BCR.ABL* negative patients.

A close examination of the transcriptomic data of normal and leukemic hematopoietic cells (Supp. Figure S3), as well as of data from the literature about the six genes found here associated with ALL prognosis suggests that they are probably involved in yet unknown oncogenic mechanisms. Although the functional data available for these genes are scarce, a few hypotheses can be made.

Except *CAMSAP1*, our selected genes showed active expression levels in at least one specific hematopoietic cell type/stage. Indeed *AK022211, FASTKD1* and *PCGF6* are preferentially expressed expression in early precursors of hematopoietic cells (CD34 positive cells from cord blood and/or bone marrow), whereas *STARD4* is active in blood B-lymphocytes and *SH3RF3* in bone marrow neutrophils. Therefore, in theory, the abnormally high levels of expression that we observe could be due either to an abnormal activation of a gene or an abnormal increase in the number of cells normally expressing it. For instance, a high expression level of the genes corresponding to a particular cell type, i.e. CD34+, could correspond either to an increase in the proportions of this cell type or to a gene activation. Since very few samples showed expression of all three CD34+ predominant genes, and only a minority overexpressed two of the CD34+ genes (illustrated by the Venn diagrams in Supp Figure S7), we conclude that the abnormally high expression levels that we observe correspond most probably to deregulated gene activity in cells rather than a mere change in cell composition.

The fact that we could identify ALL sub-groups associated with a poor outcome in both children and adults allowed us to unveil their characteristic molecular profile. Interestingly, a Gene Set Enrichment Analysis (GSEA) approach showed that, in both cases, a gene expression program characterizing embryonic stem cells is down regulated while genes defining hematopoietic stem cells are over-expressed. A close inspection of these gene sets suggests that, while these aggressive tumors keep a hematopoietic stem-like profile, they turn down the proliferative nature of the embryonic stem cells (see Figure 4D and its legend for details).

From these analyses it appears that the less responsive forms of ALL could actually be those with less proliferation and therefore less likely to respond as well to agents that target the cell cycle.

Altogether, this work demonstrates the power of an approach based on the detection of aberrant gene expression to find new prognostic factors, thereby improving existing risk based classification. This strategy is especially relevant in that it appears to detect high-risk patients currently misclassified by existing criteria to low/standard risk groups. Finally, the ability to stratify early in treatment may enable changes in therapy to reduce tumor burden quickly during induction as opposed to waiting for later MRD based time points.

## MATERIALS AND METHODS

The transcriptomic analysis, detection of aberrant expressions, assessment of prognostic associations, characterization of aggressive ALL expression profile and Gene Set Enrichment Analysis, and RT-qPCR detections, were performed as described before [1] and are detailed below.

### Meta-analysis of available transcriptomic data

All the analysed data were available from the GEO website (http://www.ncbi.nlm.nih.gov/geo/) in Affymetrix technology (54K: **Affymetrix Human Genome U133 Plus 2.0 Array**).

The following datasets were used.

-Expression in normal human tissue samples: GSE3526 for non-germline adult tissues, GSE7434, GSE9994 and GSE18809 for placenta, GSE9440 for ES, and GSE15431, GSE6872 and GSE6969 for male germ cells.

-Expression in normal hematopoietic cells: GSE11092 and GSE19735 for Cord blood cells, GSE3526 for total bone marrow, lymph nodes, spleen and tonsils, GSE12662 for CD34 positive cells, promyelocytes and neutrophils (PMN) of bone marrow, GSE9119 for resting and activated blood B cells, and GSE13159 for 74 samples of non-leukemic bone marrows from adult patients, which included healthy bone marrow specimens and nonleukemia conditions, such as megaloblastic anemia, hemolysis, iron deficiency, or idiopathic thrombocytopenic purpura [24].

-Expression in two children ALL series: GSE11877 (207 cases; [12]) and GSE7440 (59 cases; [13]). It is of note that all 207 patients included in the GSE11877 study were high-risk B-precursor ALL patients, and were treated uniformly with a modified augmented Berlin-Frankfurt-Munster (BFM) regimen [12].

-Expression in a large adult ALL series (MILES study, GSE13159; *n* = 2096 cases; 750 cases of ALL bone marrows) ([24, 25]).

All the above transcriptomic data were obtained with in Affymetrix Human Genome U133 Plus 2.0 Array technology.

-Expression in another series of adult ALL (GSE34861) [26, 27]. Since the data from this series were obtained in a different technology (GPL15088: NimbleGen Human Expression Array), the normalized data were used, and the controls consisted in the three non-leukemic samples from the same study.

Firstly, the pattern of expression of all human genes was assessed in normal tissues. The aim was to define a list of genes never expressed in normal bone marrow. A strategy similar to that used in [1] was applied here, but with a particular aim at defining genes not expressed in bone marrow tissue. We considered the transcriptomic data from various normal human tissues samples available online from publicly accessible databases (data GEO references detailed above).

All these data were available on the GEO database (http://www.ncbi.nlm.nih.gov/geo/). For the Affymetrix dat, the CEL files were downloaded and the data were normalized using R software (version 2.10.0) with the RMA algorithm for summarization and quantile normalization. These normalized expression values were then used to establish a list of genes with a predominant expression in one tissue and, for each of these genes, to identify the group of tissues in which the maximum expression was found according to the following method. Tissues were pooled into four defined groups, germline cells (including male and female germline cells), embryonic stem cells (ES), placenta, and adult somatic tissues. For each gene, the values corresponding to the mean expression in all somatic adult tissues and the standard deviation were calculated, as well as the maximum of the expression values. The group of tissues where this maximum expression was found was also recorded. A gene was defined as predominantly expressed in a tissue group if its maximum of expression was above a threshold defined as the mean value of the signals in all normal somatic tissues + 3x standard deviation. Otherwise, the gene was considered with no particular specificity of expression. A first list of genes with predominant expression other than in normal adult somatic tissues was established, encompassing genes with a highest level of expression in germline or embryonic stem (ES) cells or placenta (Figure 1: 1^st^ step), which represented approximately a third of the genome.

In order to detect aberrant gene expression, we defined a threshold value for each gene. Considering each one of the germline/ES/placenta genes, this threshold value was defined according to the distribution of expression signal values in a subset of 112 normal adult somatic tissues (from GSE3526). This threshold corresponded to the mean signal in the 112 control tissues + 3 standard deviations. After calculating the threshold value of each gene, we tested for their expression in each of the 74 samples of non-leukemic bone marrows (from GSE13159), and all genes with an expression signal above the threshold, even sporadically in a few of the non-leukemic bone marrows, were filtered out. This step enabled us to ensure that the expression of each gene was always low in non-leukemic bone marrows.

These same threshold values were then used to discriminate between expression and no expression of the genes in blast from blood or bone marrow of patients. Importantly the data of the adult and children ALL series, respectively referenced as GSE13159 and GSE11877, were obtained in the same Affymetrix technology and the corresponding .CEL files were downloaded and normalized together with the normal adult somatic tissue samples.

For the data of GSE 34861, the threshold values were calculated by a similar approach but using the non-leukemic samples from this same study.

### Assessment of prognostic values of the abnormal expression of germline/ES genes in the bone marrow of children ALL patients

Each of the germline/ES genes found expressed in at least 10% of the children ALL patients of both series GSE11877 and GSE7440 was tested for its prognostic potential in a univariate analysis, using R software (version 2.10.0). The overall survival probabilities were compared between the patients whose leukemic blasts expressed the gene (signal over the threshold established as described above) and those whose blasts did not. A logrank Mantel-Cox test allowed comparing the two survival curves. For each gene, hazard ratios (HR) for the association with the overall survival probability were calculated independently in both settings. Out of the 2119 genes, 416 were consistently associated with shorter survival in both studies (HR > 1), whereas 693 were consistently associated with longer survival in both studies (HR < 1). Respectively 6 and 17 out of the genes negatively or positively associated with prognosis had a logrank test *p*-value < 0.05 in both studies. The first three of each list were retained. These genes were then tested in combinations in univariate and multivariate approaches in the two childhood ALL series (GSE11877 and GSE7440).

We first ranked the patients according to the total number of 3 aberrantly expressed genes (detected in their bone marrow blasts or peripheral blood) associated with good prognosis and compared their survival probabilities. Supp. Figure S4A (left panel) shows an increase of the survival probability with increasing numbers of expressed genes. The patients were classified into two subgroups defined according to the number of expressions of these 3 genes positively correlated with prognosis (Supp. Figure S4A: right panel). Patients expressing 2 or more of the 3 genes were assigned to group “A pos”, with the most favorable prognosis. Patients expressing none or only one of these genes (group “B pos”) had a lower survival rate.

A similar approach using the 3 genes associated with poor prognosis showed that the patients expressing none of the 3 genes had a much higher survival probability than the patients expressing one or more of these genes (Supp. Figure S4B: left panel). The patients were also classified into two groups defined according to the number of expressions of the 3 genes negatively correlated with prognosis (Supp. Figure S4B: right panel). The patients with no expression of any of these 3 genes, with the best prognosis, were assigned to the “A neg” group, and those whose bone marrow expressed at least one of the four genes were assigned to the poor prognosis “B neg” group.

Then the patients were classified using both combinations of 3 and 3 genes, respectively associated with good and poor survival, and the survival probabilities of the four different groups were compared (Supp. Figure S4C: left panel). As expected, the patients whose bone marrow expressed 2 or more of the 3 genes positively associated with good survival and none of genes associated with poor prognosis had a high survival chance (“AA” or “P1” group). On the contrary, the 61 patients expressing at least one of the poor prognostic genes and also expressing none or only one of the good prognosis genes (“BB”) clearly had a much poorer outcome than the other patients, suggesting that their ALL was highly aggressive and with a poor response to therapy. Such patients were assigned to the poor prognosis group “P3”. The patients remaining in the intermediate group, “P2” (encompassing “AB” and “BA” patients), had a survival probability, which was not very different from the P1 patients and therefore P1 and P2 patients were all finally assigned to a good prognosis group “P1&2” (Supp. Figure S4C: right panel).

In order to examine the discriminating power of the 6 genes in combination in the context of other parameters influencing ALL outcome, we also performed sensitivity analyses considering each of the subgroups of patients which were associated with prognosis.

The global survival over 5 years was additionally modelled using a multivariate proportional hazard (Cox) model. The proportional hazard assumption was checked for each regression using the Schoenfeld's test. Nine explanatory variables were considered, which included gender, age (dichotomized < 10 or > 10years old), number of circulating white blood cells at diagnosis (WBC, dichotomized < 50G/ml or > = 50G/ml), presence of leukemic blasts in central nervous system (CNS), *MLL* status, MRD at day 29 (MRD_d29), mutation or deletion of *IKZF1* (*IKZF1*mutdel), over-expression of *CRLF2* (*CRLF2* expression), as well as the prognostic classes defined by our combination of 6 genes (P3vsP1&2). A univariate regression of the survival data was first performed against each of the nine variables. Four variables had a log-rank *p*-value below 20%, and were included in the multivariate analysis: *MLL*, MRD day29, *CRLF2* over-expression and P3vsP1&2. The following approaches were applied in order to determine which groups of variables had a joint significant contribution: backward elimination using the Akaike criterion (AIC), the BIC criterion (BIC), and forward completion using stepwise anova. The regressions of survival data against P3vsP1&2, MRD at day29 and *CRLF2* expression individually, then against all pairs, then against all three combined, were computed and compared as embedded models using Fisher's test of analysis of variance. Finally, the optimal model was tested against its completion by the interaction of the two variables.

### Supervised transcriptomic analyses

The genes showing significant differences of expression between patients with best prognosis (P1&2) and those with poorest prognosis (P3) were identified using a Mann-Whitney unpaired test (threshold *p* < 0.01). The Heatmap was obtained by representing the RMA normalized values of expression of these genes in the indicated color scale (green: low expression; red: high expression) using the permutMatrix software (version 1.9.3).

### Gene set enrichment analysis (GSEA)

The whole list of gene probes represented on the technology “Affymetrix.GeneChip.HG-U133_Plus_2”, associated with the fold changes corresponding to their differential expression between P3 and P1&2 tumors was entered as a pre-ranked file for GSEA analysis, to systematically look for significant overlaps within the gene sets database, MSigDB, made available by the Broad Institute (Molecular Signatures Database: http://www.broadinstitute.org/gsea/msigdb/index.jsp).

The Weighted Kolmogorov Smirnov test (Charmpi and Ycart, http://arxiv.org/abs/1410.1620) was used. Compared to the classical GSEA method, both use a cumulated weight function as an enrichment score, but the WKS test centers and rescales it, leading to a computation of *p*-values which is both more precise and more efficient in computing time.

### Adult ALL patients and therapeutic induction protocol

A total of 62 adult ALL patients were included in a prospective study between December 2010 and July 2014. The diagnostic and classification were performed according to the 2001 WHO (World Health Organization) classification criteria (available in [28]), which included a proportion of lymphoblasts in bone marrow > 25% and morphological, immunophenotypical and cytogenetic specific characteristics. Molecular analyses were also carried out (see Table S7 for more details).

All patients received a modified CALGB induction chemotherapy (VDLCP regimen), [16, 21] administered over a 4-week period, including:

V: vincristine 1.4mg/m2, up to 2mg per dose, intravenously, once a week for 4 weeks;
D: daunorubicin 60mg/m2 or idarubicin 8-10mg/m2, days 1 to 3;
C: cyclophosphamide 750mg/m2, days 1 and 14;
P: prednisone 60mg/m2/day, days 1 to 21;
L-asparaginase: 6000 IU/m2, every 2 days was added on days 8 to16.

Induction failure was defined as the persistence of leukemic blasts in the bone marrow (marrow with more than 5% blasts) or leukemic blasts found at an extramedullary site at the end of the induction therapy. Complete remission was defined as marrow with < 5% blasts, and normal cellular bone marrow with trilineage hematopoiesis.

The measurement of minimal residual disease (MRD) [29] was performed by eight-colour flow cytometry, as fully described in [30].

The ethical board of the Ruijin hospital of Shanghai approved this study. All patients gave their informed consent for both the treatment and cryopreservation of their bone marrow (BM) and peripheral blood samples according to the Declaration of Helsinki.

### RT-qPCR to detect the aberrant expression of the 6 genes in RNA samples from ALL adult patients

RNA from bone marrows were extracted using the « NucleoSpin RNA II » column kit (MACHEREY-NAGEL, ref740955), which included a 20 min DNAse digestion, recovered in 35 μl of DEPC/H2O, and the concentration was evaluated using a Nanodrop. One μg of total RNA (depending on availability) was reverse transcribed in a final volume of 20μl using the “SuperScript III First Strand Synthesis” kit (INVITROGEN, ref11752250). For “no RT” controls, the same volume of mix was added to the RNA without the reverse transcriptase enzyme. In each Quantitative polymerase chain reaction (qPCR), 5 μl of a 1/50 dilution of the RT product were mixed with 10μl of “Brilliant III Ultra Fast SYBR Green QPCRMaster Mix” (AGILENT, ref 600883) and 150 nM of each primer (the primer sequences are listed in Supp. Table S8) in a 20 μl final volume. The qPCR were carried out on a Stratagene Mx3000P system in 96-well plates as follows: denaturation for 3 min at 95°C, followed by 40 cycles of 20 s at 95°C and 20 s at 60°C, finally followed by a last cycle of 20 s at 95°C, 10 s at 60°C and 20 s at 95°C (to obtain the dissociation curve). Each experiment was performed in duplicates, included RT, and “no RT” samples. For each pair of primers the efficiency coefficient was calculated based on the Ct obtained at different concentrations of RNA from Testis/Placenta (since all 6 genes were expressed in male germline cells and/or placenta) and the dissociation curves were checked. When “no RT” experiments were satisfactory (Ct > = 36), for each RT experiment, using the mean Ct value of the duplicates (in all our experiments duplicates were less than 0.5 Ct apart), an expression value was calculated (2^(Ct of gene of interest in testis – Ct of gene of interest in sample))/(2^(mean Ct of the 4 control genes in testis – mean Ct of the 4 control genes in sample)), and expressed as the ratio of expression relative to testis. The four control genes were *Actin, U6, RELA, AUP1*. These 4 housekeeping genes, *Actin, RELA, AUP1, U6*, showed comparable levels of amplifications in all tested samples, since their coefficients of variation were ranged between 5% and 10% of their Ct values. In addition to the 6 prognostic genes, 3 genes, *COX8C, DKFZp761D1918* and *RPL10L,* were also detected, which are frequently abnormally expressed in ALL blasts, although not correlated with favorable or poor prognosis (corresponding primers listed in Supp. Table S8). The purpose is to help assessing the quality of the samples, in particular when none of the prognostic genes are expressed, by confirming that abnormally expressed genes can be detected.

Seven normal bone marrow samples and three cord blood samples were used to determine a threshold of aberrant expression (corresponding to the mean expression value + two standard deviations of these 10 samples). A gene was considered positively expressed when its expression value was found above this threshold.

## SUPPLEMENTARY MATERIAL FIGURES AND TABLES


