# Supplementary material for: A six gene expression signature defines aggressive subtypes and predicts outcome in childhood and adult acute lymphoblastic leukemia
Source: Oncotarget. 2015 May 12;6(18):16527–42. doi: 10.18632/oncotarget.4113 (PMC4599287; doi:10.18632/oncotarget.4113)
Supplement: Supplementary file 1 [file oncotarget-06-16527-s001.pdf]

A six gene expression signature defines aggressive subtypes and predicts outcome in childhood and adult acute lymphoblastic leukemia

Supplementary Material

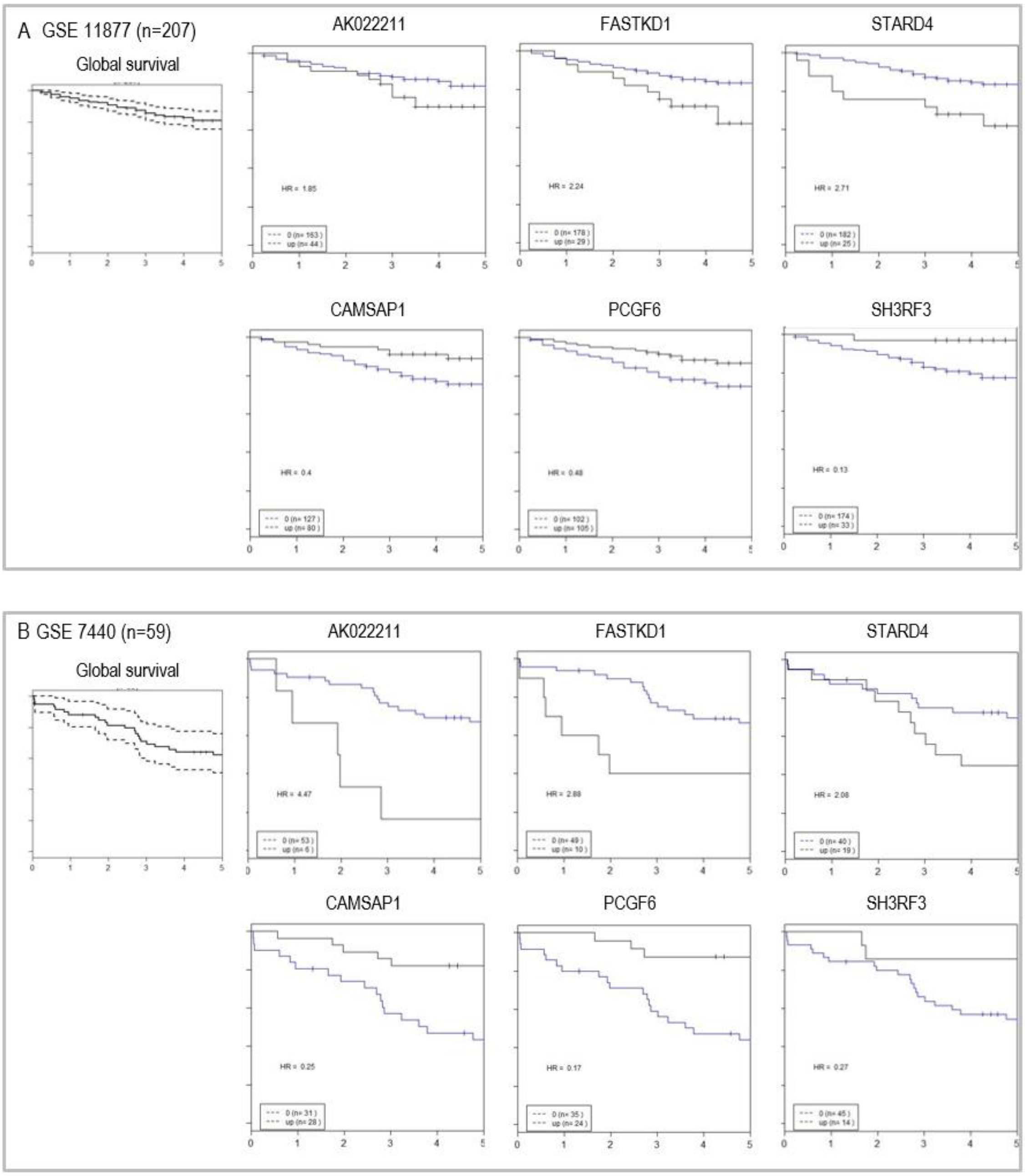

Supp. Fig 1. The expression of 6 germline/ES/placenta genes is associated with either good (A) or poor (B) prognosis in children ALL from two studies (GSE11877 & GSE7440)

For each indicated gene, the cumulative global Kaplan-Meyer survival estimates (y-axis) is shown as a function of time over five years (months; x-axis). It compares the survival probabilities of patients sub-classified into two groups according to the expression of the gene in their blood or bone marrow (not expressed: blue; expressed: black).

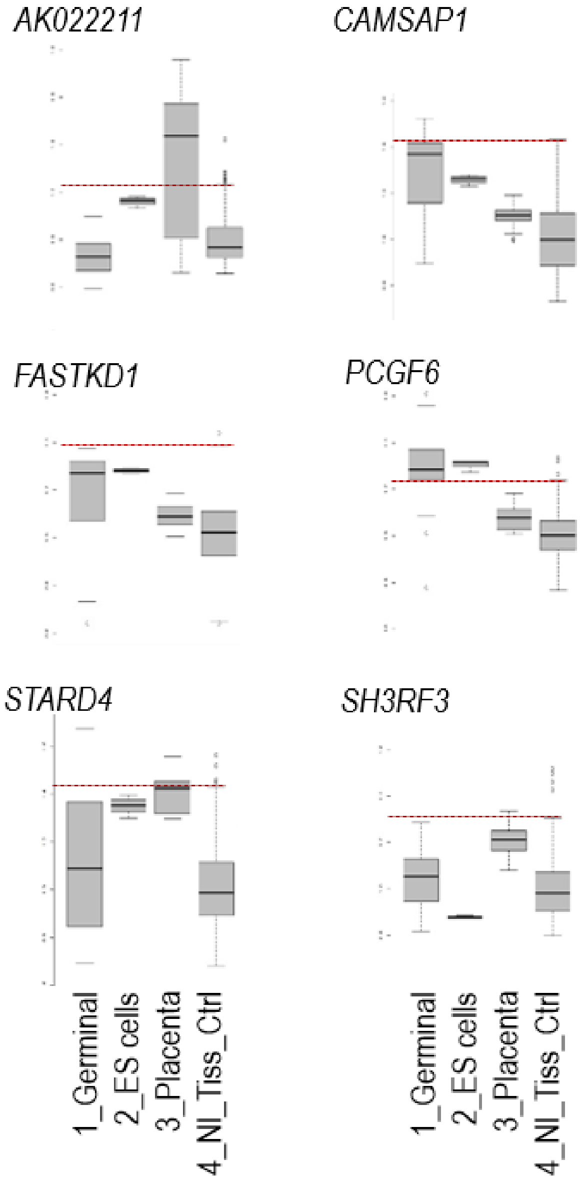

Supp. Fig 2. Expression (y-axis) of the 6 germline/ES/placenta genes in normal tissue samples grouped as follows (x-axis): "Germinal": male and female germinal cells; "ES cells": Embryonic Stem cells; "Placenta": placenta; "Nl_Tiss_Ctrl": Adult somatic tissues; The red bar was placed at the 95th percentile of the values of signals in adult somatic tissues.

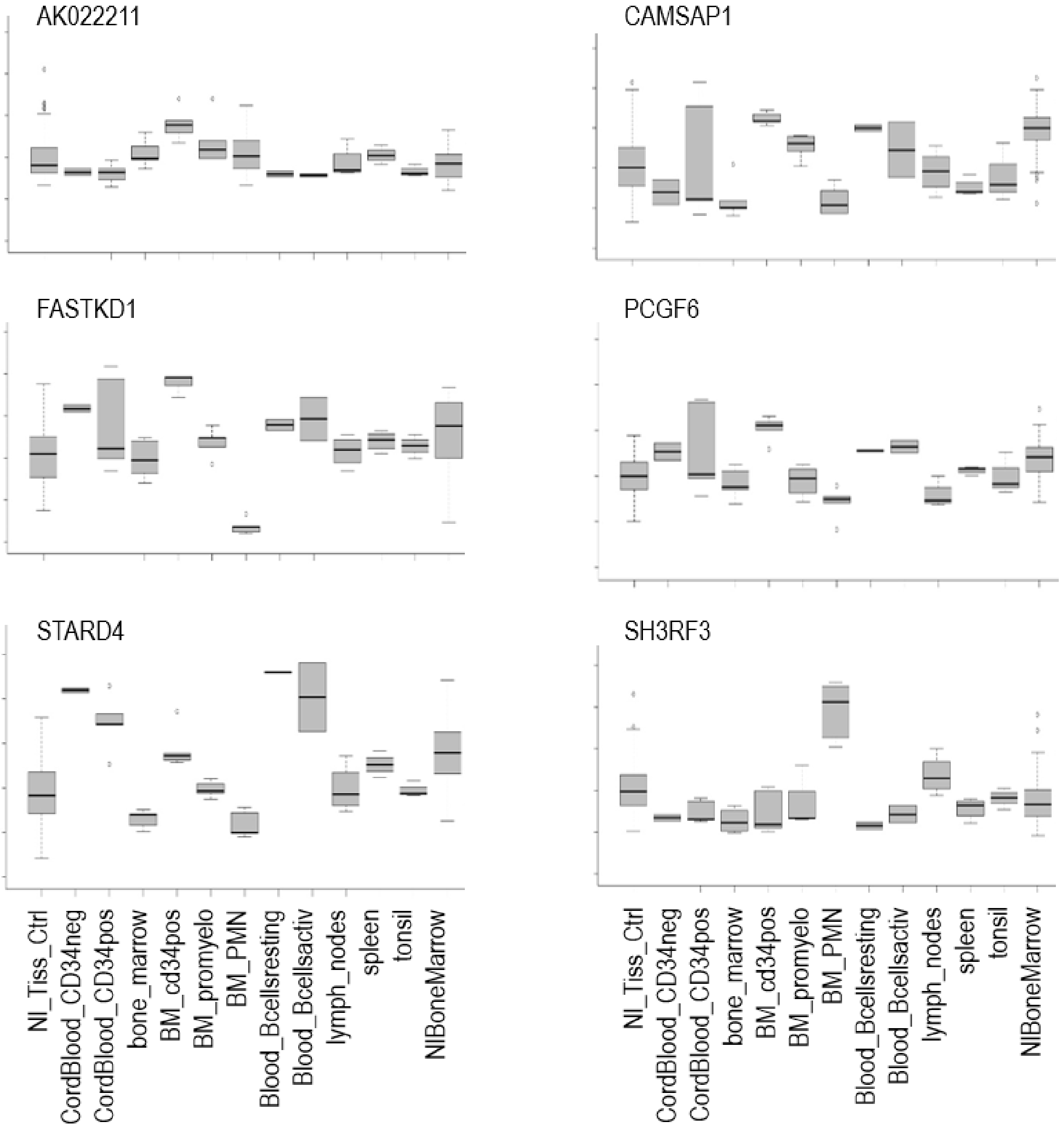

Supp. Fig 3. Expression (y-axis) of the 6 germline/ES/placenta genes in normal hematopoietic cells as follows (x-axis): "Nl_Tiss_Ctrl": Adult somatic tissues (excluding hematopoietic tissues); "CordBlood_CD34neg": CD34 negative cord blood cells; "CordBlood_CD34pos": CD34 positive cord blood cells; "Bone_marrow": Bone Marrow; "BM_cd34pos": CD34 positive cells from bone marrow; "BM_promyelo": promyelocytes from bone marrow; "BM_PMN": CD34 neutrophils from bone marrow; "Blood Bcellsresting": resting circulating B lymphocytes; "Blood Bcellsactiv": activated B lymphocytes; "lymph nodes", "spleen" and "tonsil", as indicated; "NlBoneMarrow" corresponds to the 74 non-leukemic bone marrows from adult patients in GSE13159, including healthy bone marrow specimens as well as non-leukemia conditions, such as megaloblastic anemia, hemolysis, iron deficiency, or idiopathic thrombocytopenic purpura {Haferlach, 2010 #47}.

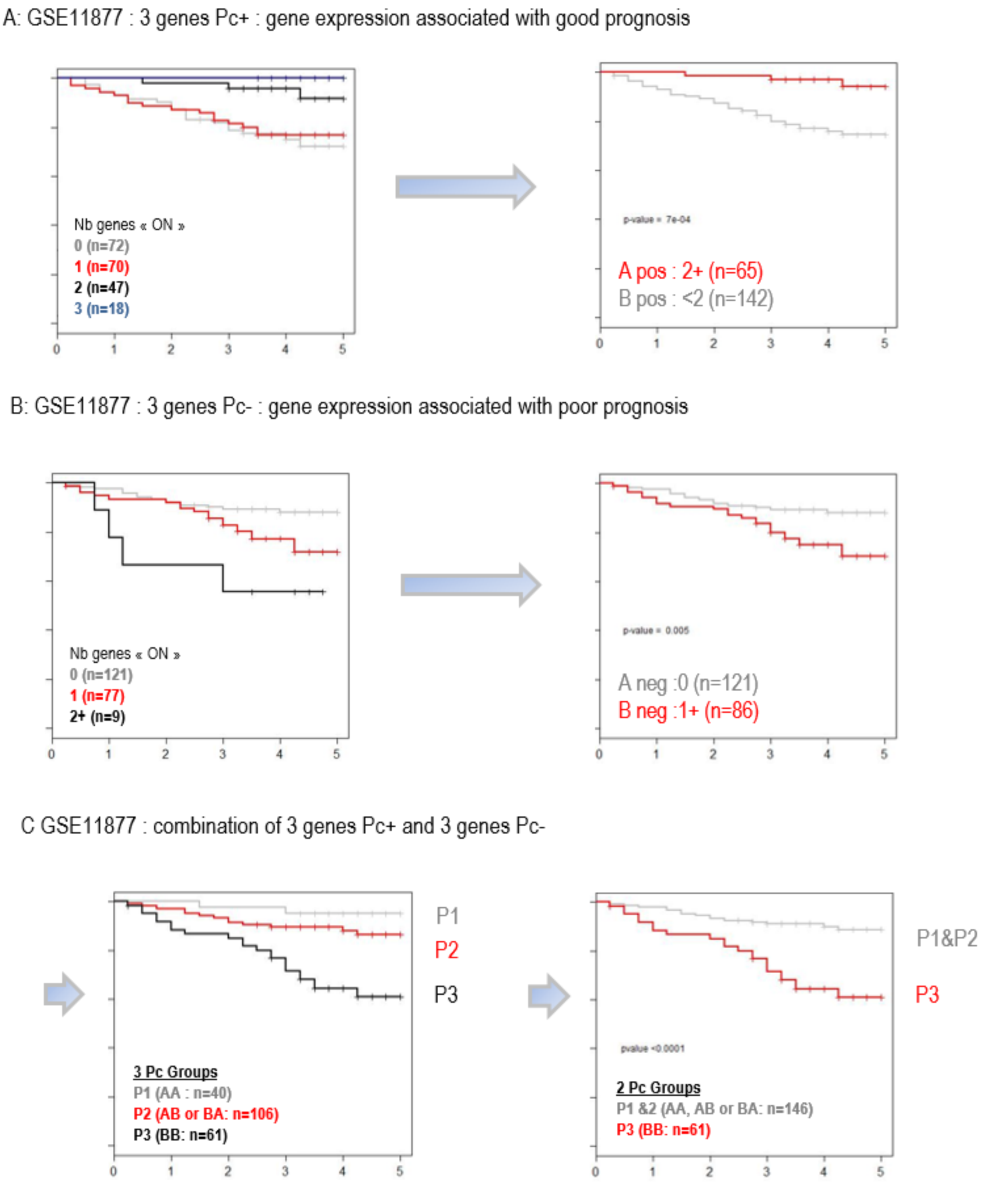

Supp. Fig 4. Definition of a classification method for the prediction of prognosis in children ALL using the expressions of combinations of 6 genes, either positively or negatively correlated with survival probabilities, and identification of a subset of aggressive ALL cases (GSE11877); **A.** Cumulative global Kaplan Meyer survival estimates comparing the patients sub-classified into groups according to the number of ectopic expressions found within the subset of 3 genes associated with good prognosis (left panel), or two groups including "A pos" (at least 2 expressed genes, red curve) or "B pos" (one or no gene expressed, grey curve) (right panel); **B.** Cumulative global Kaplan Meyer survival estimates comparing the patients sub-classified into groups according to the number of expressions found within the subset of 3 genes associated with poor prognosis (left panel), or two groups including "A neg" (0 expression, grey curve) or "B neg" (at least one expressed gene, red curve) (right panel); The A groups correspond to the patients with the highest survival probabilities, and the B groups to the patients with the lowest probability of survival. **C.** Cumulative global Kaplan Meyer survival estimates comparing the patients sub-classified into three (left panel) and two (right panel) groups according to the ectopic expressions of both sets of 3 genes associated with good or poor prognosis;

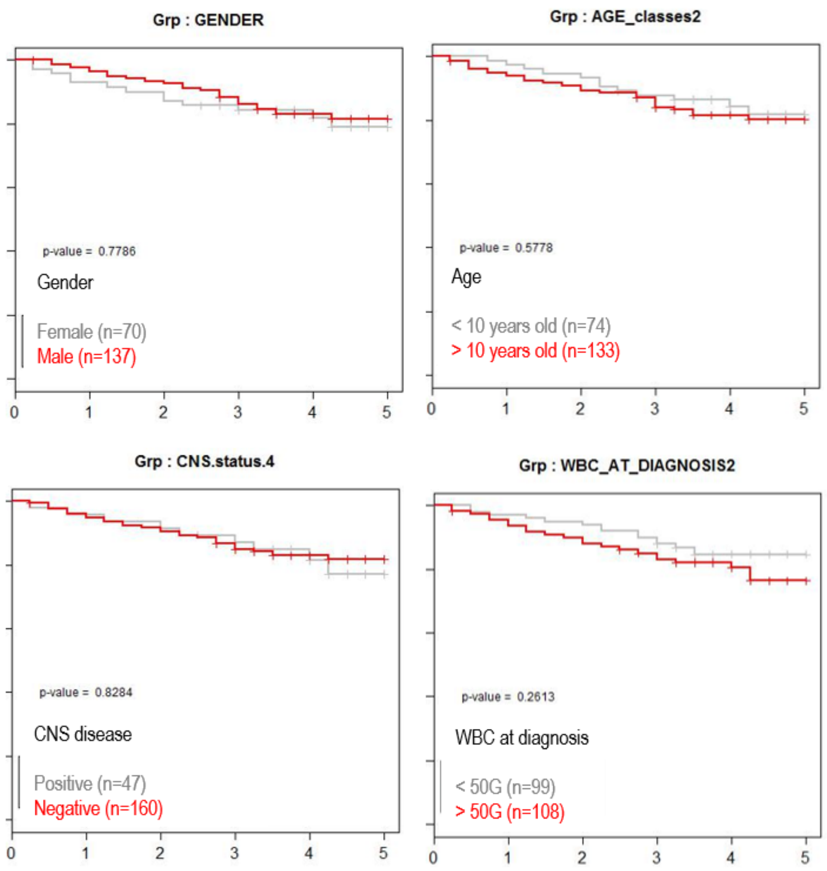

Supp. Fig. 5: Molecular/clinical classes not correlated with prognosis in this series of children ALL patients (GSE11877).

Hazard ratio of overall risk of death in P3 vs. P1&2
(GSE11877)

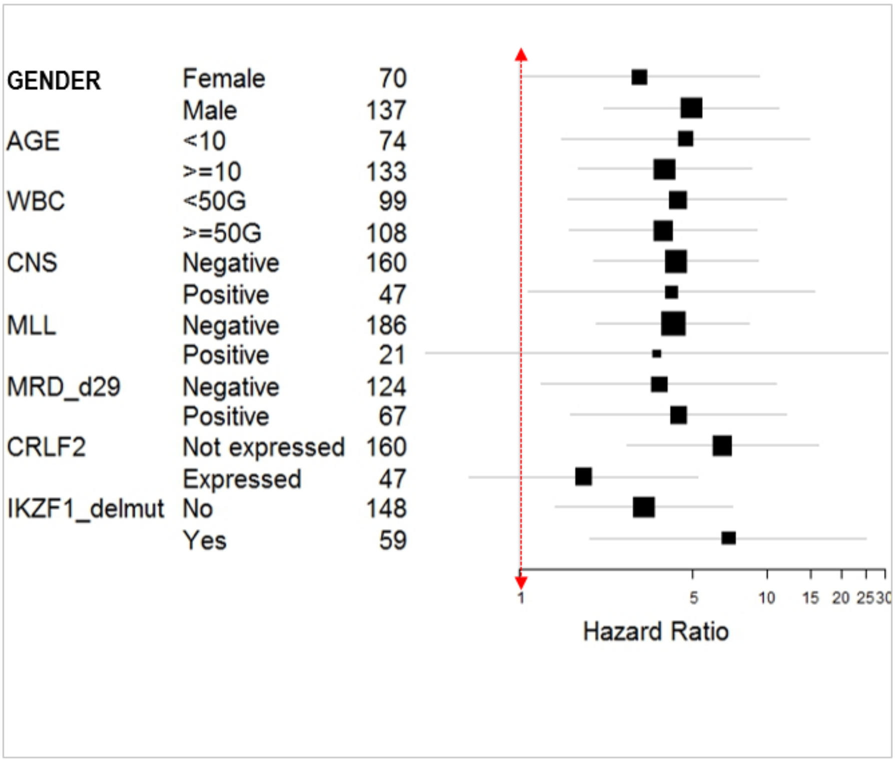

Supp. Fig. 6: Prognosis groups and bio-clinical data in children ALL (GSE11877): Forest plot of hazard ratios (P3 versus P1&2; on a log scale) for overall risk of death (over 5 years). A univariate Cox proportional hazard model estimated hazard ratios for the overall risk of death. The horizontal lines provide the 95% confidence interval for the ratios; the vertical red dotted double arrow line corresponds to a HR of 1.

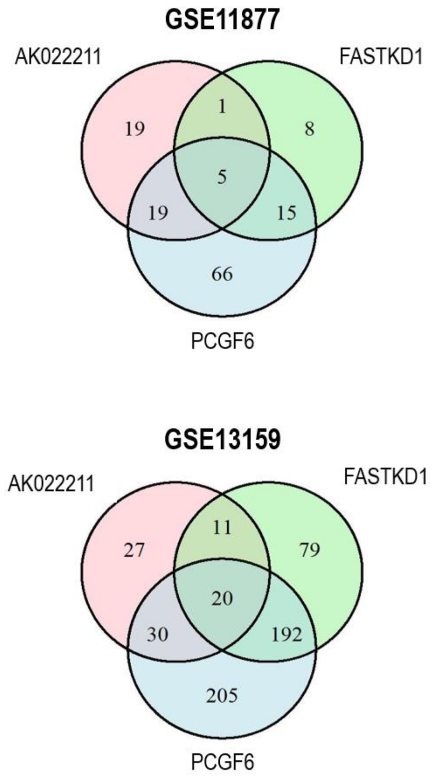

Supp. Fig. 7: Venn diagrams showing the overlaps between expressions of the three CD34 positive genes in the series of pediatric (GSE11877) and adult (GSE13159) ALL patients.

Table S1. List of 6 genes whose expression is associated with prognosis in pediatric ALL

**Gene ID**	**3 genes associated with poor prognosis in ALL**
AK022211	Homo sapiens cDNA FLJ12149 fis, clone MAMMA1000421.
FASTKD1	FAST kinase domains 1
STARD4	StAR-related lipid transfer (START) domain containing 4
	**3 genes associated with favorable prognosis in ALL**
CAMSAP1	calmodulin regulated spectrin-associated protein 1
PCGF6	polycomb group ring finger 6
SH3RF3	SH3 domain containing ring finger 3

Table S2: Modelling of the global survival over 5 years using a multivariate proportional hazard (Cox) model demonstrating that the best model includes our 6 gene classifying system (P3vsP1&2) and MRD at day 29 (MRD day29), and that both variables interact.

The proportional hazard assumption was checked for each regression using the Schoenfeld's test (PS). For GSE11877 (**A-E**), the explanatory variables considered included gender, age (dichotomized <10 or >10years old), number of circulating white blood cells at diagnosis (WBC, dichotomized <50G/ml or >=50G/ml), presence of leukemic blasts in central nervous system (CNS), *MLL* status, MRD at day 29, mutation or deletion of *IKZF1*, over-expression of the *CRLF2* gene (*CRLF2* expression), as well as the prognostic classes defined by our combination of 6 genes (P3vsP1&2).

A. GSE11877: Univariate testing of survival data against nine explaining factors. Cox proportional hazard model detects the first three as significant.

B-D. GSE11877: Multivariate testing. Starting with the model including all 3 significant factors, the backward elimination using the AIC criterion keeps all three contributing variables, P3vsP1&2, MRD at day29 and *CRLF2* expression (**B**) whereas the more stringent BIC criterion only retains our 6-genes classification algorithm (P3vsP1&2) (**C**). Starting with the most significant factor P3vsP1&2, the ANOVA forward completion also retains MRD at day29 (**D**).

E GSE11877: Interaction between variables P3vsP1&2 and MRD day29. The contingency table shows a clear dependence between P1&2vsP3 and MRD day29 (chi-squared test P=0.0003385). The reason for the detected dependence
is clear when comparing the second column with the two others, the odds of P3 for positive are significantly larger (Fisher test P= 8.115e-05). However, the Cox model of regression of the survival data against P3vsP1&2, MRD at day 29, and their interaction, is not significantly more informative than the regression against P3vsP1&2 and MRD day29 without interaction (P=0.128).

For GSE7441 (**F**), GSE34861 (**G-I**), and our series of Chinese ALL adult patients (**J-N**), the indicated molecular factors were included in a similar univariate and multivariate approach, and the informative outcome of the analyses are shown. All our multivariate models demonstrate that the most significant factor is our prognostic classes defined by the six genes combination.

A- Univariate (GSE11877)

	Pvalues	HR	HRlb	HRub	PS
**P3vsP1&2**	0.0000	4.1661	2.1580	8.0431	0.3126
**MRD day29**	0.0017	3.4591	0.2891	0.5485	0.5512
***CRLF2* expression**	0.0276	2.1783	1.1207	4.2340	0.1266
***MLL* status**	0.1888	1.8779	0.7832	4.5029	0.3216
***IKZF1* deletion or**	0.2271	1.5186	0.7814	2.9515	0.2836
**WBC**	0.2609	1.4526	0.7533	2.8013	0.6572
**Age**	0.5724	1.2167	0.6112	2.4219	0.3925
**Gender**	0.7799	0.9078	0.4622	1.7829	0.1874
**CNS**	0.8296	1.0866	0.5127	2.3028	0.4539

Pvalues	log rank test
HR	Hazard Ratio
HRlb	Hazard Ratio (lower boundary)
HRub	Hazard Ratio (upper boundary)
PS	Pvalue Schoenfeld

B- AIC backward analysis (GSE11877)

	coef	exp(coef)	se(coef)	z	p
**P3vsP1&2**	1.2	3.32	0.347	3.46	0.00055
**MRD day29 positive**	0.881	2.41	0.365	2.41	0.016
**MRD day29 unknown**	0.697	2.01	0.645	1.08	0.28
***CRLF2* expression**	0.665	1.95	0.344	1.93	0.053
Likelihood ratio test=28.4 on 4 df, p=1.02e-05 n= 207, number of events= 37					

C- BIC backward analysis (GSE11877)

	coef	exp(coef)	se(coef)	z	p
**P3vsP1&2**	1.43	4.17	0.336	4.25	2.10E-05
Likelihood ratio test=18.2 on 1 df, p=1.97e-05 n= 207, number of events= 37					

D- Anova forward analysis (GSE11877)

	loglik	Chisq	Df	Pr(>|Chi|)	
NULL	-191.63				
P3vsP1&2	-182.52	18.2182	1	1.97E-05	***
MRD day29	-179.17	6.7102	2	0.03491	*
CRLF2 expression	-177.41	3.5094	1	0.06102	.
MLL status	-177.25	0.3201	1	0.57155	
Signif. codes: 0 '***' 0.001 '**' 0.01 '*' 0.05 '.' 0.1 ' ' 1					

E- Interaction between variables P12vsP3 and MRD day29 (GSE11877)

	**MRD day29**		
**6 gene prognostic group**	Negative	Positive	Unknown
P1	98	35	13
P3	26	32	3

F- Univariate (GSE7440)

	P values	HR	HRlb	HRub	PS
**P3vsP1&2**	0.00033	4.82265	2.00537	11.59784	0.817764
***BCR/ABL* (t(9;22))**	0.41028	1.95353	0.45417	8.402686	0.657993
***CRLF2* expression**	0.64401	0.81618	0.34233	1.945932	0.876967

G- Univariate (GSE34861)

	Pvalues	HR	HRlb	HRub	PS
**P3vsP1&2**	0.014	1.60626	1.08601	2.38E+00	0.907508
BCR/ABL	0.13951	1.29681	0.92038	1.83E+00	0.399622
***MLL* status**	0.489	0.83497	0.49508	1.41E+00	0.856446
**Cytogenetic anomaly (any)**	0.51702	1.12107	0.79251	1.59E+00	0.794129
***E2A/PBX1* (t(1;19))**	0.75252	0.83595	0.26571	2.63E+00	0.075962

H- AIC or BIC backward analysis (GSE34861)

	coef	exp(coef)	se(coef)	z	p
**P3vsP1&2**	0.474	1.61	0.2	2.37	0.018
Likelihood ratio test=6.04 on 1 df, p=0.014 n= 187, number of events= 133					

I- Anova forward analysis (GSE34861)

	loglik	Chisq	Df	Pr(>|Chi|)	
**NULL**	-621.88				
**P3vsP1&2**	-618.86	6.0386	1	0.014	*
BCR/ABL	-618.25	1.2185	1	0.2697	

J- Univariate (our series RT-QPCR)

	Pvalues	HR	HRlb	HRub	PS
**P3vsP1&2**	0.00553	3.25231	1.3073	8.091137	0.267327
**MRD day29**	0.0947	1.87775	0.88527	3.982882	0.975573
BCR/ABL	0.16353	1.71197	0.81609	3.591327	0.303792

K- AIC backward analysis (our series RT-QPCR)

	coef	exp(coef)	se(coef)	z	p
**P3vsP1&2**	1.18	3.25	0.465	2.54	0.011
Likelihood ratio test=7.7 on 1 df, p=0.00553 n= 62, number of events= 29					

L- BIC backward analysis (our series RT-QPCR)

	coef	exp(coef)	se(coef)	z	p
P3vsP1&2	1.18	3.25	0.465	2.54	0.011
Likelihood ratio test=7.7 on 1 df, p=0.00553 n= 62, number of events= 29					

M- Anova forward analysis (our series RT-QPCR)

	**loglik**	**Chisq**	**Df**	**Pr(>|Chi|)**	
NULL	-102.3				
**P3vsP1&2**	-98.448	7.6973	1	0.00553	**
**MRD day29**	-98.063	0.7711	1	0.37986	
BCR/ABL	-97.969	0.1884	1	0.66422	

N- Interaction between variables P3vsP1&2 and MRD day29 (our series RT-QPCR)

	**MRD day29**	
**P3vsP1&2**	Negative	Positive
**P1**	20	9
**P3**	11	22
X-squared = 6.4786, df = 1, p-value = 0.01092 (not independent)		

Table S3. Distribution of molecular subtypes of adult ALL within the P1&2 and P3 groups defined by our six genes algorithm.

**Types ALL**	**P1&2**	**P3**	**Total**
**ALL**	**120**	**14**	**134**
with hyperdiploid karyotype	38	2	40
with t(1;19)	33	3	36
with t(12;21)	49	9	58
**c-ALL/Pre-B-ALL**	**229**	**130**	**359**
with t(9;22)	67	55	122
wo t(9;22)	162	75	237
**mature B-ALL**	**7**	**6**	**13**
**Pro-B-ALL**	**33**	**37**	**70**
**T-ALL**	**66**	**108**	**174**
**Total**	**455**	**295**	**750**

Table S4: List of genes up or down regulated (respectively 27 and 2) in aggressive ALL (P3) compared to the P1&2 ALL with a "P3-like" (P1&2P3L) transcriptomic signature (Mann Whitney p-value <0.01 and absolute fold change >1.2).

**Gene_Symbol**	**up or down in P3 vs P1&2P3Like**
PARP8	up
FAM150B	up
CDK6	up
ETV6	up
PTEN	up
PLD1	up
MIR155HG	up
MLF1IP	up
STARD4	up
AGPS	up
CXXC5	up
ATAD2	up
LOC100506935	up
ARPP19	up
TAPT1	up
SENP6	up
SC4MOL	up
CTGF	up
DPYD	up
NFIL3	up
RGS1	up
TMED5	up
ACSL1	up
IGFBP7	up
DSTN	up
FAM91A2	up
SYTL3	up
CYB5R2	down
TBC1D9	down

Table S5: GSEA gene sets from the "C2" collections, enriched or depleted in the gene signature of aggressive ALL (P3 versus P1&2) in both GSE11877 (pediatric ALL, n=207) and GSE13159 (adult ALL patients, n=750).

genesets depleted in P3 ALL (C2)	pvalue in GSE11877	pvalue in GSE13159	pvalGSE11877xGSE13159
HADDAD_B_LYMPHOCYTE_PROGENITOR	0.00036358	0.00036358	1.3219E-07
PILON_KLF1_TARGETS_DN	0.00036358	0.00036358	1.3219E-07
DACOSTA_UV_RESPONSE_VIA_ERCC3_DN	0.00036358	0.00036358	1.3219E-07
GRAESSMANN_APOPTOSIS_BY_DOXORUBICIN_DN	0.00036358	0.00036358	1.3219E-07
BUYTAERT_PHOTODYNAMIC_THERAPY_STRESS_UP	0.00036358	0.00036358	1.3219E-07
REACTOME_IMMUNE_SYSTEM	0.00036358	0.00052778	1.9189E-07
KRIEG_HYPOXIA_NOT_VIA_KDM3A	0.000678218	0.00036358	2.46586E-07
CHARAFE_BREAST_CANCER_LUMINAL_VS_MESENCHYMAL_DN	0.000678218	0.00036358	2.46586E-07
BLALOCK_ALZHEIMERS_DISEASE_UP	0.000678218	0.00036358	2.46586E-07
SCHLOSSER_SERUM_RESPONSE_DN	0.00036358	0.000821151	2.98554E-07
ONKEN_UVEAL_MELANOMA_UP	0.00036358	0.000821151	2.98554E-07
DACOSTA_UV_RESPONSE_VIA_ERCC3_COMMON_DN	0.00036358	0.000821151	2.98554E-07
NUYTTEN_NIPP1_TARGETS_DN	0.00036358	0.001225452	4.4555E-07
DAZARD_RESPONSE_TO_UV_NHEK_DN	0.00036358	0.001482948	5.3917E-07
RODRIGUES_THYROID_CARCINOMA_POORLY_DIFFERENTIATED_DN	0.001093666	0.00052778	5.77215E-07
LINDGREN_BLADDER_CANCER_CLUSTER_1_DN	0.00036358	0.002822654	1.02626E-06
FOSTER_TOLERANT_MACROPHAGE_DN	0.003175416	0.00036358	1.15452E-06
KRIGE_RESPONSE_TO_TOSEDOSTAT_6HR_UP	0.003292228	0.00036358	1.19699E-06
GARY_CD5_TARGETS_UP	0.002103623	0.000678218	1.42671E-06
DOUGLAS_BMI1_TARGETS_UP	0.001609325	0.001093666	1.76006E-06
GENTILE_UV_HIGH_DOSE_DN	0.000678218	0.002822654	1.91437E-06
CUI_TCF21_TARGETS_2_DN	0.006134324	0.00036358	2.23032E-06
ZWANG_TRANSIENTLY_UP_BY_1ST_EGF_PULSE_ONLY	0.006468634	0.00036358	2.35187E-06
SCHAEFFER_PROSTATE_DEVELOPMENT_6HR_DN	0.001981457	0.001225452	2.42818E-06
FORTSCHEGGER_PHF8_TARGETS_DN	0.000959176	0.002822654	2.70742E-06
KOINUMA_TARGETS_OF_SMAD2_OR_SMAD3	0.00258525	0.001225452	3.1681E-06
LIAO_METASTASIS	0.00258525	0.001355084	3.50323E-06
KRIGE_RESPONSE_TO_TOSEDOSTAT_24HR_UP	0.010202964	0.00036358	3.70959E-06
PATIL_LIVER_CANCER	0.00036358	0.014731666	5.35614E-06
MARTORIATI_MDM4_TARGETS_FETAL_LIVER_DN	0.00036358	0.015694394	5.70617E-06
SHEPARD_CRUSH_AND_BURN_MUTANT_DN	0.00036358	0.017507252	6.36529E-06
GOBERT_OLIGODENDROCYTE_DIFFERENTIATION_DN	0.017826477	0.00036358	6.48135E-06
REACTOME_ADAPTIVE_IMMUNE_SYSTEM	0.001225452	0.005574682	6.83151E-06
GEORGES_TARGETS_OF_MIR192_AND_MIR215	0.00036358	0.022594357	8.21485E-06
SENGUPTA_NASOPHARYNGEAL_CARCINOMA_UP	0.00036358	0.022699929	8.25324E-06
BENPORATH_CYCLING_GENES	0.00036358	0.02312207	8.40672E-06
LASTOWSKA_NEUROBLASTOMA_COPY_NUMBER_DN	0.000821151	0.010420293	8.55664E-06
NUYTTEN_EZH2_TARGETS_UP	0.025439939	0.00036358	9.24945E-06
JOHNSTONE_PARVB_TARGETS_3_UP	0.02659659	0.00036358	9.66999E-06

SPIELMAN_LYMPHOBLAST_EUROPEAN_VS_ASIAN_DN	0.00036358	0.030058563	1.09287E-05
FULCHER_INFLAMMATORY_RESPONSE_LECTIN_VS_LPS_UP	0.030582154	0.00036358	1.11191E-05
SENESE_HDAC3_TARGETS_UP	0.000821151	0.014088561	1.15688E-05
MARSON_BOUND_BY_FOXP3_UNSTIMULATED	0.00036358	0.03225612	1.17277E-05
PUJANA_ATM_PCC_NETWORK	0.00036358	0.034763027	1.26391E-05
REACTOME_CYTOKINE_SIGNALING_IN_IMMUNE_SYSTEM	0.004671615	0.002822654	1.31864E-05
WHITFIELD_CELL_CYCLE_G2_M	0.00036358	0.039347948	1.43061E-05
NUYTTEN_EZH2_TARGETS_DN	0.00036358	0.040388189	1.46843E-05
MIYAGAWA_TARGETS_OF_EWSR1_ETS_FUSIONS_UP	0.002704198	0.005462349	1.47713E-05
DAZARD_UV_RESPONSE_CLUSTER_G6	0.000678218	0.023333055	1.58249E-05
PASQUALUCCI_LYMPHOMA_BY_GC_STAGE_DN	0.00258525	0.006357312	1.64352E-05
REACTOME_SIGNALLING_BY_NGF	0.00258525	0.006468634	1.6723E-05
LINDGREN_BLADDER_CANCER_CLUSTER_3_UP	0.00036358	0.046721321	1.69869E-05
BOYLAN_MULTIPLE_MYELOMA_C_D_UP	0.004671615	0.003640662	1.70078E-05
REACTOME_DEVELOPMENTAL_BIOLOGY	0.012152185	0.00258525	3.14164E-05
BROWNE_HCMV_INFECTION_16HR_UP	0.000821151	0.048586467	3.98968E-05
ENK_UV_RESPONSE_EPIDERMIS_DN	0.04506209	0.000959176	4.32225E-05
BENPORATH_OCT4_TARGETS	0.016548366	0.002822654	4.67103E-05
RICKMAN_METASTASIS_UP	0.017826477	0.002822654	5.0318E-05
UDAYAKUMAR_MED1_TARGETS_DN	0.026806738	0.002103623	5.63913E-05
KEGG_PATHWAYS_IN_CANCER	0.022594357	0.00258525	5.84121E-05
HAN_SATB1_TARGETS_DN	0.01515982	0.003871463	5.86907E-05
FARMER_BREAST_CANCER_BASAL_VS_LULMINAL	0.002225015	0.026386396	5.87101E-05
GRAESSMANN_RESPONSE_TO_MC_AND_DOXORUBICIN_DN	0.001609325	0.038931676	6.26537E-05
BILD_E2F3_ONCOGENIC_SIGNATURE	0.012798914	0.004898385	6.2694E-05
BORCZUK_MALIGNANT_MESOTHELIOMA_UP	0.001734429	0.037578076	6.51765E-05
DURCHDEWALD_SKIN_CARCINOGENESIS_DN	0.016548366	0.003986463	6.59694E-05
BENPORATH_NANOG_TARGETS	0.001609325	0.041843514	6.73398E-05
LU_EZH2_TARGETS_DN	0.004671615	0.01537373	7.18202E-05
GAUSSMANN_MLL_AF4_FUSION_TARGETS_C_UP	0.027856812	0.00258525	7.20168E-05
MARSON_BOUND_BY_FOXP3_STIMULATED	0.002225015	0.033196756	7.38633E-05
GOZGIT_ESR1_TARGETS_DN	0.046202948	0.001609325	7.43555E-05
BASSO_CD40_SIGNALING_UP	0.022594357	0.003524832	7.96413E-05
LINDGREN_BLADDER_CANCER_CLUSTER_2A_DN	0.009876579	0.008456155	8.35179E-05
IVANOVA_HEMATOPOIESIS_LATE_PROGENITOR	0.007465931	0.014731666	0.000109986
POOLA_INVASIVE_BREAST_CANCER_UP	0.019843978	0.005686875	0.00011285
MILI_PSEUDOPODIA_HAPTOTAXIS_DN	0.020797291	0.005910855	0.00012293
DOANE_RESPONSE_TO_ANDROGEN_DN	0.008784898	0.014624556	0.000128475
DAVICIONI_MOLECULAR_ARMS_VS_ERMS_UP	0.032465209	0.004215727	0.000136864
DACOSTA_UV_RESPONSE_VIA_ERCC3_XPCS_DN	0.003524832	0.04018019	0.000141628
STARK_PREFRONTAL_CORTEX_22Q11_DELETION_UP	0.006468634	0.022171922	0.000143422
HADDAD_T_LYMPHOCYTE_AND_NK_PROGENITOR_UP	0.043401542	0.003524832	0.000152983
BENPORATH_SOX2_TARGETS	0.005124452	0.032987784	0.000169044
PILON_KLF1_TARGETS_UP	0.027751854	0.006690953	0.000185686
ZHENG_BOUND_BY_FOXP3	0.028696099	0.007686538	0.000220574
ACEVEDO_LIVER_CANCER_UP	0.006690953	0.033092274	0.000221419
SENESE_HDAC3_TARGETS_DN	0.01814551	0.013659209	0.000247853

PID_HES_HEYPATHWAY	0.010528881	0.024071065	0.000253441
DEURIG_T_CELL_PROLYMPHOCYTIC_LEUKEMIA_DN	0.026176155	0.009985428	0.00026138
RAO_BOUND_BY_SALL4	0.016974768	0.017507252	0.000297182
BENPORATH_NOS_TARGETS	0.013229336	0.022805486	0.000301701
XU_GH1_AUTOCRINE_TARGETS_DN	0.041323888	0.007576277	0.000313081
GROSS_HYPOXIA_VIA_ELK3_UP	0.024597812	0.013766595	0.000338628
WANG_LMO4_TARGETS_UP	0.013444339	0.025755533	0.000346266
REACTOME_SIGNALING_BY_NOTCH	0.03256974	0.01063742	0.000346458
GU_PDEF_TARGETS_UP	0.023754857	0.014731666	0.000349949
BAKKER_FOXO3_TARGETS_DN	0.01920763	0.021960614	0.000421811
WHITFIELD_CELL_CYCLE_G1_S	0.010311654	0.042466877	0.000437904
WAMUNYOKOLI_OVARIAN_CANCER_LMP_DN	0.025860706	0.019949972	0.00051592
HIRSCH_CELLULAR_TRANSFORMATION_SIGNATURE_DN	0.044958345	0.011504042	0.000517203
BOSCO_ALLERGEN_INDUCED_TH2_ASSOCIATED_MODULE	0.048690041	0.01074591	0.000523219
TIEN_INTESTINE_PROBIOTICS_6HR_DN	0.026071017	0.020585565	0.000536687
WAKABAYASHI_ADIPOGENESIS_PPARG_RXRA_BOUND_8D	0.042259112	0.013014195	0.000549968
BROWNE_HCMV_INFECTION_20HR_UP	0.01708131	0.034763027	0.000593798
REACTOME_NGF_SIGNALLING_VIA_TRKA_FROM_THE_PLASMA_MEMBRANE	0.016868203	0.037786395	0.000637389
SHAFFER_IRF4_TARGETS_IN_ACTIVATED_DENDRITIC_CELL	0.017720091	0.040908081	0.000724895
CHICAS_RB1_TARGETS_SENESCENT	0.025229483	0.030058563	0.000758362
KEGG_SMALL_CELL_LUNG_CANCER	0.021432072	0.036431832	0.00078081
LINSLEY_MIR16_TARGETS	0.017720091	0.046617656	0.000826069
PEDERSEN_METASTASIS_BY_ERBB2_ISOFORM_7	0.048897177	0.016974768	0.000830018
LIU_SOX4_TARGETS_UP	0.031419421	0.032674264	0.001026606
SASAKI_ADULT_T_CELL_LEUKEMIA	0.044543314	0.030582154	0.00136223
IKEDA_MIR30_TARGETS_UP	0.049000738	0.031733248	0.001554953
KIM_MYCN_AMPLIFICATION_TARGETS_DN	0.041531756	0.038619405	0.001603932
CREIGHTON_ENDOCRINE_THERAPY_RESISTANCE_1	0.041115996	0.039972167	0.001643495
KEGG_NEUROTROPHIN_SIGNALING_PATHWAY	0.039556046	0.046721321	0.001848111
ONKEN_UVEAL_MELANOMA_DN	0.041115996	0.046410312	0.001908206
BASAKI_YBX1_TARGETS_DN	0.040388189	0.049207847	0.001987416
**genesets enriched in P3 ALL (C2)**	**pv11877l C2g**	**Pv13159l C2g**	**pval1187 7x13159**
GOZGIT_ESR1_TARGETS_DN	0.00036358	0.006357312	2.31139E-06
GRUETZMANN_PANCREATIC_CANCER_UP	0.00036358	0.010202964	3.70959E-06
MARTENS_BOUND_BY_PML_RARA_FUSION	0.000959176	0.005237239	5.02343E-06
EBAUER_TARGETS_OF_PAX3_FOXO1_FUSION_UP	0.002822654	0.002940656	8.30045E-06
BOSCO_ALLERGEN_INDUCED_TH2_ASSOCIATED_MODULE	0.00036358	0.024176441	8.79007E-06
WIERENGA_STAT5A_TARGETS_DN	0.003986463	0.003058233	1.21915E-05
REACTOME_NGF_SIGNALLING_VIA_TRKA_FROM_THE_PLASMA_MEMBRANE	0.00036358	0.036744527	1.33596E-05
CHARAFE_BREAST_CANCER_LUMINAL_VS_BASAL_DN	0.009440625	0.001482948	1.4E-05
CHYLA_CBFA2T3_TARGETS_UP	0.004671615	0.003058233	1.42869E-05
SMIRNOV_RESPONSE_TO_IR_6HR_UP	0.00052778	0.039452	2.0822E-05
JAATINEN_HEMATOPOIETIC_STEM_CELL_UP	0.001609325	0.018995363	3.05697E-05
REACTOME_HEMOSTASIS	0.001858429	0.01814551	3.37221E-05
BEIER_GLIOMA_STEM_CELL_DN	0.002940656	0.012798914	3.76372E-05
CREIGHTON_ENDOCRINE_THERAPY_RESISTANCE_3	0.005462349	0.011071096	6.04742E-05

REACTOME_SIGNALLING_BY_NGF	0.0037562	0.017932843	6.73593E-05
MARKEY_RB1_CHRONIC_LOF_UP	0.0037562	0.018995363	7.13504E-05
CHARAFE_BREAST_CANCER_LUMINAL_VS_MESENCHYMAL_DN	0.031524039	0.002940656	9.27013E-05
KRIGE_RESPONSE_TO_TOSEDOSTAT_24HR_DN	0.041219945	0.002940656	0.000121214
PILON_KLF1_TARGETS_DN	0.011287657	0.012260069	0.000138387
IVANOVA_HEMATOPOIESIS_STEM_CELL_AND_PROGENITOR	0.020691436	0.006912855	0.000143037
HOEBEKE_LYMPHOID_STEM_CELL_UP	0.0037562	0.048068524	0.000180555
KRIGE_RESPONSE_TO_TOSEDOSTAT_6HR_DN	0.039452	0.006022651	0.000237606
HUANG_GATA2_TARGETS_UP	0.021643536	0.011287657	0.000244305
REACTOME_METABOLISM_OF_LIPIDS_AND_LIPOPROTEINS	0.039035753	0.006801954	0.000265519
GOLDRATH_ANTIGEN_RESPONSE	0.009658716	0.029534734	0.000285268
LEE_DIFFERENTIATING_T_LYMPHOCYTE	0.01708131	0.017932843	0.000306316
ONO_AML1_TARGETS_DN	0.013981271	0.022171922	0.000309992
TONKS_TARGETS_OF_RUNX1_RUNX1T1_FUSION_HSC_DN	0.009440625	0.034971731	0.000330155
COULOUARN_TEMPORAL_TGFB1_SIGNATURE_UP	0.03256974	0.011828298	0.000385245
LINDGREN_BLADDER_CANCER_CLUSTER_2B	0.013444339	0.029115497	0.000391439
CHEMNITZ_RESPONSE_TO_PROSTAGLANDIN_E2_DN	0.020903129	0.018889201	0.000394843
ONO_FOXP3_TARGETS_DN	0.021432072	0.024176441	0.000518151
KOINUMA_TARGETS_OF_SMAD2_OR_SMAD3	0.011395871	0.048482887	0.000552505
BROWNE_HCMV_INFECTION_14HR_DN	0.040388189	0.014410248	0.000582004
ACOSTA_PROLIFERATION_INDEPENDENT_MYC_TARGETS_DN	0.016228314	0.040596164	0.000658807
BROWNE_HCMV_INFECTION_18HR_DN	0.027016842	0.031314794	0.000846027
WAKABAYASHI_ADIPOGENESIS_PPARG_RXRA_BOUND_8D	0.037786395	0.028591225	0.001080359
MARSON_FOXP3_TARGETS_DN	0.025545149	0.046306633	0.00118291
BASAKI_YBX1_TARGETS_DN	0.028905818	0.048068524	0.00138946
LEE_TARGETS_OF_PTCH1_AND_SUFU_DN	0.04475084	0.033301229	0.001490258
KRIGE_RESPONSE_TO_TOSEDOSTAT_24HR_UP	0.043712999	0.038827592	0.00169727
MIKKELSEN_ES_ICP_WITH_H3K27ME3	0.036952956	0.047654085	0.001760959

Table S6: Gene Ontology (GO) terms enriched or depleted in the gene signature of aggressive ALL (P3 versus P1&2) in both GSE11877 (pediatric ALL, n=207) and GSE13159 (adult ALL patients, n=750).

	GOterms enriched in P3 ALL (C5)	pv11877lC5g	pv13159lC5g	pval11877x13159
Cytoskeleton and Cell adhesion	CYTOSKELETAL_PART	0.004330015	0.038827592	0.000168124
	CYTOSKELETON	0.026806738	0.015694394	0.000420716
	MICROTUBULE_CYTOSKELETON	0.017613682	0.039660085	0.00069856
	REGULATION_OF_CELL_ADHESION	0.039347948	0.021432072	0.000843308
	POSITIVE_REGULATION_OF_CELL_ADHESION	0.033823477	0.031314794	0.001059175
Metabolic process	LIPID_METABOLIC_PROCESS	0.0174008	0.049311394	0.000858058
Proliferation	HOMEOSTASIS_OF_NUMBER_OF_CELLS	0.015587532	0.046617656	0.000726654
	INTERPHASE	0.049725539	0.044958345	0.002235578
Immune system	ADAPTIVE_IMMUNE_RESPONSE_GO_0002460	0.027121876	0.039972167	0.00108412
	ADAPTIVE_IMMUNE_RESPONSE	0.031733248	0.034763027	0.001103144
Cell signalling	NEGATIVE_REGULATION_OF_SIGNAL_TRANSDUCTION	0.047239571	0.021220544	0.001002449

	GO terms depleted in P3 ALL (C5)	pv11877gC5g	pv13159gC5g	pval11877x13159
Nuclear Regulations	TRANSCRIPTION_FACTOR_BINDING	0.00036358	0.008126775	2.95473E-06
	TRANSCRIPTION	0.00036358	0.012260069	4.45751E-06
	REGULATION_OF_GENE_EXPRESSION	0.00036358	0.014945799	5.43399E-06
	REGULATION_OF_TRANSCRIPTION	0.00036358	0.01537373	5.58958E-06
	NEGATIVE_REGULATION_OF_TRANSCRIPTION	0.001482948	0.004444086	6.59035E-06
	NUCLEUS	0.00036358	0.03705716	1.34732E-05
	TRANSCRIPTION_COFACTOR_ACTIVITY	0.00036358	0.038515302	1.40034E-05
	TRANSCRIPTION_DNA_DEPENDENT	0.00036358	0.042778482	1.55534E-05
	REGULATION_OF_TRANSCRIPTIONDNA_DEPENDENT	0.00036358	0.047135931	1.71377E-05
	DNA_BINDING	0.000821151	0.040076182	3.29086E-05
	TRANSCRIPTION_ACTIVATOR_ACTIVITY	0.004444086	0.042466877	0.000188726
	NEGATIVE_REGULATION_OF_TRANSCRIPTION_DNA_DEPENDENT	0.022488771	0.014731666	0.000331297
	TRANSCRIPTION_REPRESSOR_ACTIVITY	0.042051325	0.021220544	0.000892352
	TRANSCRIPTION_COREPRESSOR_ACTIVITY	0.030372745	0.032465209	0.000986058
	POSITIVE_REGULATION_OF_TRANSCRIPTION	0.025965868	0.038202953	0.000991973
	NEGATIVE_REGULATION_OF_TRANSCRIPTION_FROM_RNA_POLYMERASE_II_PROMC	0.048275715	0.041843514	0.002020026
Metabolic process	NEGATIVE_REGULATION_OF_CELLULAR_METABOLIC_PROCESS	0.00036358	0.004444086	1.61578E-06
	NEGATIVE_REGULATION_OF_METABOLIC_PROCESS	0.00036358	0.00534987	1.94511E-06
	NUCLEOBASENUCLEOSIDENUCLEOTIDE_AND_NUCLEIC_ACID_METABOLIC_PROCESS	0.00036358	0.006801954	2.47305E-06
	BIOPOLYMER_METABOLIC_PROCESS	0.00036358	0.009003726	3.27357E-06
	NEGATIVE_REGULATION_OF_NUCLEOBASENUCLEOSIDENUCLEOTIDE_AND_NUCLEIC	0.001093666	0.003524832	3.85499E-06
	REGULATION_OF_CELLULAR_METABOLIC_PROCESS	0.00036358	0.01430305	5.2003E-06
	REGULATION_OF_NUCLEOBASENUCLEOSIDENUCLEOTIDE_AND_NUCLEIC_ACID_MET	0.00036358	0.016974768	6.17168E-06
	REGULATION_OF_METABOLIC_PROCESS	0.00036358	0.019843978	7.21487E-06
	PHOSPHOTRANSFERASE_ACTIVITY_ALCOHOL_GROUP_AS_ACCEPTOR	0.009767676	0.032360669	0.000316089
	CELLULAR_MACROMOLECULE_METABOLIC_PROCESS	0.010854352	0.032151563	0.000348984
	POSITIVE_REGULATION_OF_NUCLEOBASENUCLEOSIDENUCLEOTIDE_AND_NUCLEIC_	0.019843978	0.034449913	0.000683623
	RNA_BIOSYNTHETIC_PROCESS	0.00036358	0.043090037	1.56667E-05
	PROTEIN_METABOLIC_PROCESS	0.006245876	0.023016556	0.000143759
	TRANSFERASE_ACTIVITY_TRANSFERRING_PHOSPHORUS_CONTAINING_GROUPS	0.008126775	0.042778482	0.000347651
	NEGATIVE_REGULATION_OF_RNA_METABOLIC_PROCESS	0.026071017	0.017720091	0.000461981
Proliferation	CELL_PROLIFERATION_GO_0008283	0.001225452	0.002345709	2.87455E-06
	REGULATION_OF_CELL_PROLIFERATION	0.014624556	0.031942423	0.000467144
Cell signalling	KINASE_ACTIVITY	0.006022651	0.032046997	0.000193008
	MAPKKK_CASCADE_GO_0000165	0.042051325	0.039660085	0.001667759
Cellular processes	NEGATIVE_REGULATION_OF_BIOLOGICAL_PROCESS	0.000959176	0.001609325	1.54363E-06
	CELLULAR_PROTEIN_METABOLIC_PROCESS	0.009222298	0.030058563	0.000277209
	NEGATIVE_REGULATION_OF_CELLULAR_PROCESS	0.000959176	0.001858429	1.78256E-06
	POSITIVE_REGULATION_OF_CELLULAR_PROCESS	0.001355084	0.010528881	1.42675E-05
	POSITIVE_REGULATION_OF_BIOLOGICAL_PROCESS	0.003986463	0.003640662	1.45134E-05

Table S7. Clinical and biological data of our 62 ALL patients and detailed results of our 6-genes RT-qPCR based detection test.

ID	AGE	SEX	WBC counts($x10^9$)	Immunophenotype	t(9;22)/BCR-ABL	blast infiltration in BM	MRD Day 29	induction response (CR=early response; NR= no response)	PFS_month	OS_month	Status_Dead (1) versus Alive (0)	Status_Relapse(1) vs remission(0)	AK022211	FASTKD1	STARD4	CAMSAP1	PCGF6	SHR3F3	COX8C	DKFZ	RPL10L	Nb of negative genes_up	Nb of positive genes_up	6 genes prognostic class P1P2P3	6 genes prognostic class P1&2 VERSUS P3
S1	35	F	26	B-ALL	pos	0.42	pos	NR	10	17	0	1	0	1	1	0	0	0	0	0	0	2	0	P3	P3
S11	40	F	32	B-ALL	neg	0.92	pos	CR	3	5	1	1	0	0	0	0	1	1	1	1	1	0	2	P1	P1&2
S12	22	M	15	B-ALL	neg	0.87	neg	CR	35	36	0	0	0	0	0	0	0	0	1	0	0	0	0	P2	P1&2
S13	65	M	60	T-ALL	neg	0.97	pos	NR	0	4	1	1	0	0	1	0	0	0	0	0	0	1	0	P3	P3
S14	38	M	3	TandB-ALL	neg	0.79	dead	dead	0	0.5	1	1	0	0	0	0	1	0	1	1	0	0	1	P2	P1&2
S15	54	F	1	T-ALL	neg	0.80	pos	NR	3	4	0	0	0	0	0	0	1	0	1	1	1	0	1	P2	P1&2
S16	56	F	28	B-ALL	neg	0.88	pos	NR	0	3	1	1	0	1	0	0	1	0	1	1	0	1	1	P3	P3
S17	17	M	37	T-ALL	neg	0.90	neg	CR	28	30	0	0	0	0	1	0	0	0	1	0	1	1	0	P3	P3
S18	34	F	1	B-ALL	neg	0.75	neg	CR	3	4	0	0	0	0	0	0	1	1	1	1	0	0	2	P1	P1&2
S19	28	M	249	B-ALL	pos	0.90	pos	NR	3	14	1	1	0	1	0	0	0	0	1	1	0	1	0	P3	P3
S2	18	M	62	B-ALL	neg	0.96	neg	CR	15	17	0	0	0	0	0	0	0	0	1	1	0	0	0	P2	P1&2
S21	56	F	26	B-ALL	neg	0.88	pos	NR	0	4	0	1	0	1	1	0	0	0	1	0	0	2	0	P3	P3
S22	59	M	53	T-ALL	neg	0.91	pos	NR	8	20	0	1	0	0	0	0	0	0	0	0	0	0	0	P2	P1&2
S24	36	F	286	B-ALL	pos	0.78	pos	NR	0	5	1	1	0	1	1	0	1	0	0	0	0	2	1	P3	P3
S25	59	M	5	B-ALL	pos	0.65	neg	CR	3	4	0	0	0	0	0	0	0	0	1	0	1	0	0	P2	P1&2
S26	59	F	7	B-ALL	pos	0.48	neg	CR	2	4	0	0	0	0	0	1	0	1	1	0	1	0	2	P1	P1&2
S27	15	F	4	B-ALL	neg	0.63	pos	CR	4	5	0	0	0	0	0	0	0	0	1	0	0	0	0	P2	P1&2
S28	38	M	41	B-ALL	neg	0.84	neg	CR	6	12	1	1	0	0	0	0	1	0	1	0	0	0	1	P2	P1&2
S29	20	M	53	B-ALL	pos	0.89	pos	NR	0	11	1	1	0	1	0	0	1	0	1	0	1	1	1	P3	P3
S3	24	M	84	B-ALL	pos	0.95	pos	NR	6	9	1	1	0	1	0	0	0	0	1	1	1	1	0	P3	P3
S33	27	F	1	B-ALL	neg	0.72	neg	CR	5	7	0	0	0	1	0	1	0	1	1	0	0	1	2	P2	P1&2
S36	53	F	303	B-ALL	neg	0.72	neg	CR	4	5	0	0	1	0	0	0	1	0	1	0	0	1	1	P3	P3
S37	15	F	16	B-ALL	neg	0.94	neg	CR	3	4	0	0	0	0	0	1	1	0	1	1	0	0	2	P1	P1&2
S38	59	F	60	B-ALL	pos	0.93	neg	CR	2	2	1	1	0	0	1	0	1	0	1	0	0	1	1	P3	P3
S39	15	M	7	B-ALL	neg	0.96	neg	CR	13	15	1	1	0	1	0	0	1	0	0	0	1	1	1	P3	P3

S4	60	F	67	B-ALL	pos	0.52	pos	NR	0	1	1	1	0	1	1	0	0	0	1	0	1	2	0	P3	P3
S40	22	M	439	B-ALL	pos	0.95	pos	CR	2	3	0	0	0	1	0	1	0	1	1	0	0	1	2	P2	P1&2
S41	42	F	135	B-ALL	pos	0.92	neg	CR	3	6	1	1	0	0	0	1	1	1	1	1	0	0	3	P1	P1&2
S42	16	M	34	B-ALL	neg	0.97	neg	CR	3	4	0	0	0	0	0	0	0	0	1	1	0	0	0	P2	P1&2
S43	27	F	14	T-ALL	neg	0.92	dead	dead	0	1	1	1	0	0	1	0	0	0	0	0	0	1	0	P3	P3
S44	26	M	37	B-ALL	neg	0.89	neg	CR	2	3	0	0	0	0	0	1	1	1	1	1	1	0	3	P1	P1&2
S45	39	F	43	B-ALL	neg	0.80	pos	CR	3	4	0	0	0	0	0	0	1	0	1	1	1	0	1	P2	P1&2
S46	26	M	26	B-ALL	pos	0.83	neg	CR	2	3	0	0	0	0	0	0	1	0	1	1	1	0	1	P2	P1&2
S48	31	M	6	B-ALL	neg	0.86	neg	CR	42	42	0	0	0	0	1	0	0	0	0	1	1	1	0	P3	P3
S5	44	M	17	B-ALL	neg	0.94	pos	NR	0	8	1	1	0	1	0	0	0	0	1	1	1	1	0	P3	P3
S50	34	F	3	B-ALL	neg	0.65	pos	CR	6	7	0	0	0	1	0	1	1	0	1	1	0	1	2	P2	P1&2
S54	49	M	49	B-ALL	neg	0.77	pos	NR	3	3	1	1	0	1	0	0	0	0	0	0	0	1	0	P3	P3
S55	60	F	4	B-ALL	neg	0.79	pos	NR	5	6	0	0	0	1	0	0	0	0	1	0	0	1	0	P3	P3
S56	22	M	3	T-ALL	neg	0.98	pos	CR	42	42	0	0	0	0	1	0	1	0	0	1	1	1	1	P3	P3
S58	56	F	7	B-ALL	pos	0.77	dead	dead	0	0.5	1	1	0	0	0	0	0	1	0	0	0	0	1	P2	P1&2
S59	24	M	48	T-ALL	neg	0.56	pos	NR	0	9	1	1	0	0	1	0	0	0	0	0	0	1	0	P3	P3
S6	18	M	10	B-ALL	neg	0.75	neg	CR	3	4	0	0	0	0	0	1	1	1	1	1	0	0	3	P1	P1&2
S60	32	M	12	B-ALL	pos	0.57	pos	CR	12	13	0	0	0	1	0	0	1	0	1	0	1	1	1	P3	P3
S61	56	F	66	B-ALL	pos	0.97	pos	CR	16	22	1	1	0	1	0	0	0	0	0	0	0	1	0	P3	P3
S62	20	M	70	T-ALL	neg	0.74	pos	CR	6	7	0	0	0	0	1	0	1	0	0	0	0	1	1	P3	P3
S63	51	M	25	B-ALL	pos	0.80	neg	CR	3	4	0	0	0	0	0	0	1	1	1	0	0	0	2	P1	P1&2
S64	29	M	53	T-ALL	neg	0.91	pos	CR	5	6	0	0	0	0	1	0	0	0	1	0	0	1	0	P3	P3
S65	15	M	50	B-ALL	neg	0.80	neg	CR	33	33	0	0	0	0	0	0	0	0	1	1	1	0	0	P2	P1&2
S67	55	M	47	B-ALL	neg	0.91	dead	dead	0	0	1	1	0	1	0	0	0	0	1	0	0	1	0	P3	P3
S68	18	F	40	B-ALL	neg	0.95	neg	CR	4	5	0	0	0	0	0	0	1	1	1	0	0	0	2	P1	P1&2
S69	31	M	63	B-ALL	pos	0.94	pos	CR	26	28	0	0	0	1	0	0	1	0	1	0	0	1	1	P3	P3
S7	17	M	29	T-ALL	neg	0.87	neg	CR	33	33	0	0	0	1	1	0	1	0	0	1	1	2	1	P3	P3
S70	24	M	65	T-ALL	neg	0.97	pos	NR	0	3	0	1	0	1	0	0	1	0	0	1	1	1	1	P3	P3
S72	16	M	14	B-ALL	pos	0.88	pos	NR	0	12	1	1	0	0	1	0	0	0	1	0	0	1	0	P3	P3
S73	22	F	7	B-ALL	neg	0.91	pos	CR	9	9	0	0	0	0	0	0	1	1	1	0	1	0	2	P1	P1&2
S75	46	F	115	B-ALL	neg	0.91	neg	CR	2	3	0	0	0	0	0	0	1	0	0	1	1	0	1	P2	P1&2
S76	20	F	414	B-ALL	neg	0.91	neg	CR	10	11	1	1	1	0	0	0	0	0	1	0	0	1	0	P3	P3
S77	42	M	59	T-ALL	neg	0.82	neg	CR	7	9	0	0	0	0	1	1	1	0	1	1	1	1	2	P2	P1&2
S78	38	M	460	T-ALL	neg	0.98	dead	dead	0	0	1	1	0	1	1	0	1	0	0	0	0	2	1	P3	P3
S79	39	M	151	B-ALL	pos	0.96	pos	NR	18	20	1	1	0	1	0	0	0	0	0	0	0	1	0	P3	P3
S8	33	F	379	B-ALL	neg	0.97	neg	CR	6	25	1	1	0	1	1	0	1	0	1	0	0	2	1	P3	P3
S80	27	M	38	B-ALL	neg	0.74	pos	CR	2	3	0	0	0	0	0	1	0	1	1	1	0	0	2	P1	P1&2

Table S8. List of primers used for the 6-genes RT-qPCR based detection test.

Gene	**Forward primer**	**Reverse primer**
COX8C	tacttggcctcacctcacct	cacgcaggagaggcatatcg
DKFZp761D1918	aaccccaccagctatttgcc	tgctggaggagccagtagtt
RPL10L	gtgccaacaaatacatggtga	tgacatggaagggatggag
STARD4	agaagggcttttatcttgtgga	ccaaccacagggatggttat
FASTKD1	aagaattaacttttctgcatttcca	cagaacagacacctcagttggt
AK022211	acaggcttcttttcccccag	cacgcaccccttagaactgt
PCGF6	gaggacatgagtcacttctcgtt	tggggtcagctcagagagat
CAMSAP1	ccggaagaacctgaatacctc	tggcaataaaggatgggatg
SHR3F3	gcccatcggtgttctgtc	ggagcccactcttcttctcttt
PCDH15	acgcttcacacaggaggaata	gtccctttggtggcaagtt
ENSG245479	tggtgtgtagtctgactggc	tcgctctgacaccacgattt
MKI67	tcaaggaactgattcaggagaag	gtgcactgaagaacacatttcc
